# Spatial and temporal variability of soil N_2_O and CH_4_ fluxes along a degradation gradient in a palm swamp peat forest in the Peruvian Amazon

**DOI:** 10.1111/gcb.15354

**Published:** 2020-10-09

**Authors:** Kristell Hergoualc’h, Nelda Dezzeo, Louis V. Verchot, Christopher Martius, Jeffrey van Lent, Jhon del Aguila‐Pasquel, Mariela López Gonzales

**Affiliations:** ^1^ Center for International Forestry Research (CIFOR) Lima Peru; ^2^ Venezuelan Institute for Scientific Research (IVIC) Caracas Venezuela; ^3^ Center for International Tropical Agriculture (CIAT) Cali Colombia; ^4^ Center for International Forestry Research (CIFOR) Bonn Germany; ^5^ Department for Soil Quality Wageningen University & Research Wageningen The Netherlands; ^6^ Instituto de Investigaciones de la Amazonia Peruana (IIAP) Iquitos Peru

**Keywords:** GHG emissions, *Mauritia flexuosa* swamp forests, methane, nitrous oxide, peatland, Peru, tropical

## Abstract

*Mauritia flexuosa* palm swamp, the prevailing Peruvian Amazon peatland ecosystem, is extensively threatened by degradation. The unsustainable practice of cutting whole palms for fruit extraction modifies forest's structure and composition and eventually alters peat‐derived greenhouse gas (GHG) emissions. We evaluated the spatiotemporal variability of soil N_2_O and CH_4_ fluxes and environmental controls along a palm swamp degradation gradient formed by one undegraded site (Intact), one moderately degraded site (mDeg) and one heavily degraded site (hDeg). Microscale variability differentiated hummocks supporting live or cut palms from surrounding hollows. Macroscale analysis considered structural changes in vegetation and soil microtopography as impacted by degradation. Variables were monitored monthly over 3 years to evaluate intra‐ and inter‐annual variability. Degradation induced microscale changes in N_2_O and CH_4_ emission trends and controls. Site‐scale average annual CH_4_ emissions were similar along the degradation gradient (225.6 ± 50.7, 160.5 ± 65.9 and 169.4 ± 20.7 kg C ha^−1^ year^−1^ at the Intact, mDeg and hDeg sites, respectively). Site‐scale average annual N_2_O emissions (kg N ha^−1^ year^−1^) were lower at the mDeg site (0.5 ± 0.1) than at the Intact (1.3 ± 0.6) and hDeg sites (1.1 ± 0.4), but the difference seemed linked to heterogeneous fluctuations in soil water‐filled pore space (WFPS) along the forest complex rather than to degradation. Monthly and annual emissions were mainly controlled by variations in WFPS, water table level (WT) and net nitrification for N_2_O; WT, air temperature and net nitrification for CH_4_. Site‐scale N_2_O emissions remained steady over years, whereas CH_4_ emissions rose exponentially with increased precipitation. While the minor impact of degradation on palm swamp peatland N_2_O and CH_4_ fluxes should be tested elsewhere, the evidenced large and variable CH_4_ emissions and significant N_2_O emissions call for improved modeling of GHG dynamics in tropical peatlands to test their response to climate changes.

## Graphical abstract

Over 3 years of monitoring, Peruvian Amazon *Mauritia flexuosa* palm swamp peatlands showed significant N_2_O emissions and large CH_4_ emissions, the later exhibiting an exponential rise with increased precipitation. Their degradation, which results from cutting whole palms for fruit extraction, induced microscale (hummock and hollow of live and cut palms) changes in N_2_O and CH_4_ emission trends and controls but no obvious changes at the macroscale. Monthly and annual emissions were mainly controlled by variations in: WFPS, water table level (WT), and net nitrification for N_2_O; WT, air temperature and net nitrification for CH_4_.

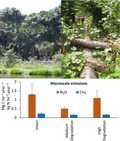

## 1 INTRODUCTION

Peatlands are characterized by their unique ability to accumulate and store organic matter over thousands of years under anaerobic conditions created by water saturation (Joosten & Clarke, 2002). These immense terrestrial carbon (C) pools are long‐term net sinks of carbon dioxide (CO_2_), large sources of atmospheric methane (CH_4_) and therefore play important roles in the global C cycle (Gorham, 1991; Lähteenoja, Ruokoleinen, Schulman, & Oinonen, 2009). Peatlands also host a unique biodiversity (Posa et al., 2011) and regulate water cycles (Bullock & Acreman, 2003). The largest areas of tropical peatlands are located in South America, Southeast Asia and the central Congo Basin (Dargie et al., 2017; Gumbricht et al., 2017). In the Peruvian Amazon, peatlands lie primarily in the Pastaza‐Marañon Basin, where they cover 3.6 M ha, harbor peat deposits up to 7.5 m thick and store around 3.1 Pg C, that is, almost half the aboveground biomass C stock of Peruvian forests (Asner et al., 2014; Draper et al., 2014; Lähteenoja & Page, 2011). Lähteenoja and Page (2011) reported a substantial diversity of peatland ecosystems in the region, from rain‐fed nutrient‐poor ombrotrophic systems to river‐fed nutrient‐rich minerotrophic swamps. The majority (80%) support a growth of *Mauritia flexuosa*‐dominated forests that are regularly flooded by dynamic rivers such as the Amazon and their tributaries (Draper et al., 2014).

Peruvian Amazonian peatlands have been partially protected from deforestation and fires in contrast to their Southeast Asian counterparts (Lilleskov et al., 2018; Roucoux et al., 2017), but have suffered intensive large‐scale degradation over decades (Horn et al., 2018). In a 350,000 ha area in the Pastaza‐Marañon Basin, 31% of *M. flexuosa* palm swamp peatlands were found to be strongly degraded, 42% moderately degraded and 27% had low levels of degradation (Hergoualc’h et al., 2017). The fruits of *M. flexuosa* (locally named *Aguaje*) are highly demanded in the local market but are often collected by cutting down the entire palm. This degradation translates into changes in vegetation cover and composition, reductions in biomass C stocks and litter inputs, and could potentially result in destabilization of peat deposits (Bhomia et al., 2019; Hergoualc’h et al., 2017; van Lent et al., 2019). Research on greenhouse gas (GHG) emissions from tropical peatlands under natural or disturbed conditions has mostly been conducted in Southeast Asia, and very little is known about fluxes in neotropical peatlands (e.g., Hoyos‐Santillan et al., 2016; Teh et al., 2017; Wright et al., 2011, 2013) and their response to ecosystem degradation. In addition, due to the preponderance of CO_2_ in drained peatlands GHG budgets, there has been continuing research emphasis on this gas despite the high global warming potential of CH_4_ and nitrous oxide (N_2_O; Myhre et al., 2013) and the need for full GHG accounting for climate change predictions (Hergoualc’h & Verchot, 2014; Sjögersten et al., 2014). Soil–atmosphere exchanges of both CH_4_ and N_2_O in peatland forests can be highly spatially variable because of microscale variations in topography. The peat surface consists of a mosaic of hummocks densely packed with roots around tree bases and sparsely vegetated hollows (Jauhiainen et al., 2005). Differences in hydrological and substrate conditions between these two microtopographies lead to large spatial variation in GHG fluxes (Jauhiainen et al., 2005). Degradation of palm swamp peatlands may alter CH_4_ and N_2_O emissions in several ways. Changes in vegetation density and composition can prompt a modification in the hummock to hollow ratio and site‐scale proportion of microtopography‐derived emissions. Vegetation changes also induce alterations in litter quantity and quality (Hergoualc’h, Hendry, et al., 2017; van Lent et al., 2019), thereby influencing GHG fluxes. Finally, degradation may also be expected to modify microclimate conditions.

Understanding mechanisms and main factors driving soil–atmosphere exchanges of CH_4_ and N_2_O is essential for constraining non‐CO_2_ budgets and for predictions of climate and land‐use changes (Aini et al., 2015; Hergoualc’h & Verchot, 2014). N_2_O fluxes from peat soils are governed by variables that limit the microbial processes of nitrification and denitrification, such as availability of mineral N and labile organic matter, soil moisture and aeration status, and soil temperature (Jauhiainen et al., 2012; Schlesinger, 2013). Soil N_2_O emissions often correlate well with the soil water‐filled pore space (WFPS), with emission rates in the tropics found to be maximum around a WFPS of 60% and to remain high at 80% WFPS (van Lent et al., 2015). CH_4_ dynamics in waterlogged soils result from the balance between CH_4_ production and consumption by soil microorganisms (Bridgham et al., 2013). Production occurs with the anaerobic decay of organic material in the soil saturated zone under highly reduced conditions. Consumption, that is, the oxidation of CH_4_ to CO_2_ mostly occurs where oxygen is available and as CH_4_ moves through less reduced zones in the peat (Horwath, 2007; Strack et al., 2008). Therefore, CH_4_ emissions in peatlands have been found to be particularly related to the water table level (WT), and several studies have reported a decline in net CH_4_ flux accompanying a lowering of the WT (Bridgham et al., 2013; Hoyos‐Santillana et al., 2016). Other general controls on wetlands CH_4_ emissions include soil temperature and vegetation (Turetsky et al., 2014). In particular, the CH_4_ production rate is influenced by C substrate quality (Hoyos‐Santillana et al., 2016; Le Mer & Roger, 2001; Wright et al., 2011, 2013). Because the biosphere reacts to climate change, the importance of the response of GHG fluxes to temperature and precipitation has received attention in past decades (Singh et al., 2010). Disruption of GHG dynamics in palm swamp peatlands may occur under future changing climate (Wang et al., 2018). Projections in the northwestern Amazon indicate a warmer climate by the end of the 21st century and a trend toward increased precipitation, and a higher frequency of wet days and floods (Barichivich et al., 2018; Espinoza et al., 2016; Gloor et al., 2013; Marengo et al., 2018). Process‐based modeling of the response of peat C accumulation rate to warmer and wetter climate suggests that palm swamp peatlands in the Peruvian Amazon may switch from a C sink to a source (Wang et al., 2018), but data uncertainty and scarcity leave doubts. For instance, CH_4_ fluxes simulated by Wang et al. (2018) were not validated against field measurements, presumably owing to unavailability of long‐term data. Also, the model focused on the C cycle disregarding any assessment of the response of N_2_O emissions to climate change.

This study investigated soil fluxes of N_2_O and CH_4_ and their environmental controls along a gradient of degradation in a peat swamp forest in the Peruvian Amazon. The gradient consisted of one undegraded site and two sites with different levels of degradation. The measurements were conducted monthly over 3 years that include El Niño and La Niña episodes. We addressed the following research questions: (a) How do N_2_O and CH_4_ fluxes vary spatially at the microscale and macroscale under undegraded and degraded conditions? (b) How do the fluxes vary intra‐annually and inter‐annually? (c) How do environmental variables control the spatial and temporal variation of the fluxes? Spatial variability at the microscale was assessed by differentiating hummocks supporting live or cut palms from surrounding hollows. The macroscale analysis considered structural changes in vegetation and soil microtopography as impacted by degradation.

## 2 MATERIALS AND METHODS

### 2.1 Site description

The study was carried out in the province of Loreto, southwest of Iquitos, in the Northern Peruvian Amazon. Based on 1948–1994 weather data, the climate of the region is warm and humid with mean annual temperature of 27°C and mean annual rainfall of 3,087 mm (Marengo, 1998). Most months (66%) exhibit precipitation rates in the range 100–300 mm; while months with precipitation either <100 mm (7%) or >400 mm (10%) are infrequent. High precipitation months (>300 mm) are infrequent between June and September, and more frequent between January and April.

This research was conducted in an area of peat swamp forest dominated by *M. flexuosa* palms, located near the Itaya River, one of the tributaries of the Amazon River (van Lent et al., 2019). Peat deposits up to 5 m deep have been reported in this forest (Lähteenoja, Ruokoleinen, Schulman, & Oinonen, 2009). Following successional ecological stages, the *M. flexuosa* swamp palm forest which persists today started to form about 1,000 years ago, with its current vegetation community established c. 400 years ago (Roucoux et al., 2013). The vegetation development over time has been highly influenced by the flooding regime. The swamp is quasi‐permanently waterlogged and occasionally floods, such as in 1998 (30 cm) and 2012 (100 cm; Roucoux et al., 2013). The peat was classified as minerotrophic (nutrient‐rich; Lähteenoja, Ruokolainen, Schulman, Alvarez, et al., 2009; van Lent et al., 2019) and likely receives nutrients during flooding events, as well as during the annual flood pulses of the Amazon River.

Along this swamp forest complex, we selected a site that was undegraded, thereafter referred to as “Intact” (S 03°49.949′ W 073°18.851′), a site moderately degraded (“mDeg,” S 03°50.364′ W 073°19.501′), and a heavily degraded site (“hDeg,” S 03°48.539′ W 073°18.428′). The Intact site was located within the protected Quistococha Regional Reserve. The mDeg and hDeg sites were adjacent to the reserve and used by local communities for extraction of *M. flexuosa* fruits and timber harvesting. The Intact site exhibited a closed canopy; the mDeg site had reduced canopy closure, and the hDeg site had a very open canopy with few standing trees (van Lent et al., 2019). *M. flexuosa* was present at all sites and was the most important species at the Intact and mDeg sites according to IVI (Importance Value Index), while the hDeg site was dominated by *Cecropia membranacea*, a pioneer tree species (Bhomia et al., 2019). The density (individuals with a diameter at breast height ≥10 cm) of *M. flexuosa* palms was 170, 164 and 16 ha^−1^ at the Intact, mDeg and hDeg sites, respectively, and that of dicotyledonous trees was 1,496, 700 and 679 ha^−1^, respectively. Peat depth and pH of stagnant water at the Intact, mDeg and hDeg sites were 2.2, >2.7, 1.0 m and 5.9, 5.9, 6.6, respectively (Bhomia et al., 2019).

### 2.2 Experimental design and GHG flux measurement

**FIGURE 1:**
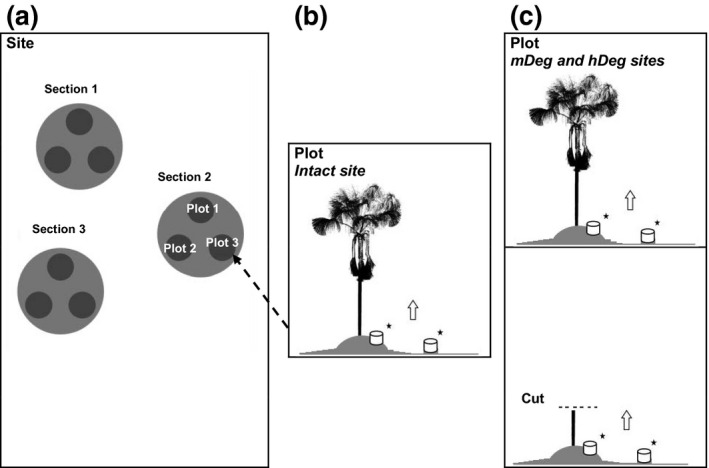
Experimental design at the study sites. (a) Layout of sections and plots within each site. (b) Layout of the subplot within a plot at the Intact site. (c) Layout of the two subplots within a plot at the medium degraded (mDeg) and highly degraded (hDeg) sites, including a standing live palm and a cut palm. Chambers were permanently installed on hummocks and hollows to measure soil CH_4_ and N_2_O fluxes. Source of the *Mauritia flexuosa* scan in (b) and (c) is from Caballero (2017)

The experiment was installed in April 2014 at the Intact site, in July 2014 at the hDeg site and in February 2015 at the mDeg site. At each site, we established three 250 m^2^ measurement sections, separated by ~150 m (Figure 1). Within each section, we set up three plots around 15 m distant from each other. At the Intact site, a plot consisted of one 2.5 × 2.5 m subplot that included a live *M. flexuosa* palm growing on a hummock (live‐hummock) and its adjacent hollow (live‐hollow). At the degraded sites, a plot consisted of two subplots: One with a live *M. flexuosa* palm similarly to the Intact site, the other with the stump of a cut *M. flexuosa* palm on a hummock (cut‐hummock) and its adjacent hollow (cut‐hollow). *M. flexuosa* palms are typically cut at 1 m height with the stump left on‐site while the remaining cut trunk is used to build walking paths inside the forest.

Soil flux measurements started in July 2014, October 2014 and April 2015 at the Intact, hDeg and mDeg sites, respectively, and terminated in July 2018. The overlapping period across sites (April 2015–July 2018) amounted to 40 months. CH_4_ and N_2_O fluxes were monthly measured by the static closed chamber method in opaque PVC chambers (25 cm height, 30 cm diameter) permanently inserted 3–5 cm into the ground. The measurements were systematically performed between 08:30 and 15:30 hr to avoid potential diel variation in gas fluxes. Dial fluctuations of CH_4_ fluxes were found to be negligible at the Intact site (Griffis et al., 2020). Two chambers were installed in each subplot, one on a hummock and another one on the adjacent hollow. On hummocks, the chamber was located <0.5 m from the palm trunk, in the main rooting zone. On hollows, it was placed >1.5 m from the trunk, outside the main rooting zone. Atmospheric pressure, soil and air temperature, and chamber height were recorded at the start of the measurements. Prior to sampling, chambers were manually fanned and then closed with a vented lid equipped with a center port for gas sampling. Gas samples (20 ml) were taken by syringe from the chamber headspace at 0, 10, 20 and 30 min after chamber closure and transferred to 10 ml pre‐evacuated glass vials. Vials were sealed with silicon, to prevent leakage during transportation, by air, from Iquitos to the laboratory in Lima. Samples were analyzed by gas chromatography (GC; Clarus 580 Perkin Elmer) using an electronic capture detector for analysis of N_2_O, and a flame ionization detector for analysis of CH_4_. The gases were separated on HayeSep^®^ columns at a temperature of 165°C up to 290°C, using a carrier gas (N_2_) flow of 30 ml/min. Soil respiration was measured concomitantly with fluxes of N_2_O and CH_4_ using the dynamic closed chamber method (EGM‐4, PP System; van Lent et al., in preparation).

Gas fluxes were calculated by linear regression of the change in concentration against time using the four samples. Whenever one sample had leaked or if the accumulation curve became nonlinear, the regression was based on three points (7% of the fluxes). The detection limit of the method was computed using the formula by Parkin et al. (2012) based on analytical precision of GC measurement of N_2_O and CH_4_ at ambient concentration and chamber deployment time. Mean and coefficient of variation of 35 N_2_O and CH_4_ ambient samples taken at random from monthly GC control procedure datasets amounted to, respectively, 330 ppb and 9% for N_2_O and 1,522 ppb and 1% for CH_4_. The detection limit was 6.8 g N ha^−1^ day^−1^ for N_2_O and 2 g C ha^−1^ day^−1^ for CH_4_. Following recommendations by Parkin et al. (2012) and Gilbert (1987), we report the actual measured flux values even if they fall within the detection limit band. The average monthly flux was computed per palm status and by microtopography (live‐hummock, live‐hollow, or cut‐hummock, cut‐hollow) based on nine replicates per site. Annual fluxes were computed per status—microtopography and site by linear interpolation between measurement dates for each of the 3 years when measurements overlapped across sites (year 1: from April 2015 to March 2016; year 2: from April 2016 to March 2017; year 3: from April 2017 to March 2018). Site‐scale annual fluxes calculation took into account structural changes in vegetation and soil microtopography caused by degradation. An inventory conducted at the sites showed that degradation reduced live palm density, increased cut palm density and decreased the size of hummocks (van Lent et al., in preparation). The relative proportion of live‐hummock, live‐hollow, cut‐hummock, cut‐hollow per site was computed from this inventory (Table 1) and used for site‐scale computation.

**TABLE 1:** Relative proportion of the area occupied by live palms on a hummock and adjacent hollow, and cut palms on a hummock and adjacent hollow at the Intact, moderately (mDeg) and heavily (hDeg) degraded sites. The proportions were assessed from an inventory conducted in three plots totaling 0.7 ha per site (van Lent et al., in preparation)

Status‐microtopography	Intact	mDeg	hDeg
Live‐hummock	0.15	0.08	0.02
Live‐hollow	0.85	0.79	0.59
Cut‐hummock	n.a.	0.01	0.02
Cut‐hollow	n.a.	0.12	0.38

Note

n.a., not available, the number of cut palms at the intact site was minimal.

### 2.3 Soil properties

In all, 24 samples per site (12 from hollows and 12 from hummocks) were collected from the upper 5 cm of the soil profile using a metal ring (radius = 4.5 cm; van Lent et al., 2019). Eighteen of them (nine from hollows and nine from hummocks) were oven‐dried at 60°C until constant mass and their bulk density was determined from the dry mass per ring volume. The remaining six samples (three per microtopography) were each ground, homogenized and analyzed for total C and N content by the induction furnace method (Costech EA C‐N Analyzer). Their exchangeable cations, cation exchange capacity and base saturation were determined using the standard ammonium acetate at pH 7 method (Pansu et al., 2001). Copper (Cu), manganese (Mn), zinc (Zn) and phosphorus (P) were determined by the Mehlich 3 method (Ziadi & Sen Tran, 2007). All analyses were performed by the University of Hawaii‐Hilo.

### 2.4 Environmental parameters

Daily precipitation rates were measured using a rain gauge with a tipping bucket (0.2 mm resolution, Delta Ohm HD2013R) equipped with a thermometer for hourly recording of air temperature. This climate station was situated between 1 and 3 km from the sites. Several environmental parameters were measured concurrently with soil GHG sampling. The WT relative to the soil surface was measured in a perforated PVC tube (5 cm in diameter and 1.5 m in length) installed permanently in the soil at 50 cm from each chamber. Soil temperature in the top 10 cm and air temperature were measured at the side of each chamber using a portable digital thermometer. Gravimetric soil moisture was measured from samples collected from the top 10 cm near each chamber. Samples were carefully transferred to sealed plastic bags taking care of not losing water, stored in a cool box and their wet mass weighted immediately upon return to the laboratory. Soil samples were oven‐dried at 60°C until reaching constant mass and soil moisture was determined on a dry mass basis. The soil WFPS was calculated based on the formula by Linn and Doran (1984) as the product of gravimetric soil moisture and bulk density divided with the porosity, assuming a particle density of 1.4 g/cm^3^ (Driessen & Rochimah, 1976). WPFS estimations were based on the average bulk densities from Table S1. Mean monthly and annual soil and air temperatures were computed at site scale by averaging values regardless of microtopography or palm status, given the observed general homogeneity at the microscale (data no shown). Site‐scale mean annual WT level and WFPS were calculated by considering the hummock to hollow proportions from Table 1, regardless of the palm status which did not influence the variables.

Nitrogen availability was determined by measuring inorganic N pools and the rates of net mineralization and nitrification quarterly over 2 years starting in October 2016. Nine replicate soil samples per microtopography and site were collected around live palms to a 5 cm depth using a ring sampler. Samples were stored on ice, transported to the laboratory and kept at 4°C for a maximum of 3 days until incubation. Coarse roots and litter were manually removed from the samples before incubation. Net mineralization and net nitrification were determined following the procedure of Hart et al. (1994). For each sample, 15 g subsample was analyzed for gravimetric moisture content by oven‐drying at 60°C for 48 hr. Another 15 g moist subsample was extracted in 100 ml of 2M KCl to determine inorganic N concentrations. These extracts were shaken for an hour and allowed to settle for 24 hr. A 20 ml aliquot of the supernatant was filtered and maintained at 4°C before analysis within a week. NH_4_^+^ content was analyzed using the formaldehyde method (INDECOPI, 2011) and NO_3_^−^ by the salicylic acid colorimetric method (Ayre & Roman, 1992) at the University of La Molina, Lima. A third subsample of 15 g was incubated in the dark at room temperature (maintained at 20°C with air conditioning) for 10 days and was subsequently extracted according to the procedures described above. Net mineralization was calculated as the change in inorganic N (NH_4_^+^ + NO_3_^−^) concentration; net nitrification as the change in NO_3_^−^ concentration over 10 days. Inorganic N stocks were calculated from the first extraction.

### 2.5 Statistics

The potential effects of disturbance level (i.e., site: Intact, mDeg and hDeg), status (live and cut palms), microtopography (hummock and hollow) and temporal variability (40 months; i.e., overlapping period across sites) on the fluxes of N_2_O and CH_4_ were examined using a mixed‐effects ANOVA model with four factors. Sampling month was treated as a random factor, whereas disturbance level, status and microtopography were treated as fixed effects. The design was asymmetric because not all status levels were present at all sites (there were no cut palms at the intact site), and it was unbalanced because not all combinations of treatments had nine replicates per sampling month (due to missing data). Considering these limitations, adequate degrees of freedom, mean squares and F‐ratio were calculated using the PERMANOVA routine in PRIMER v7. Although this method was specially designed for multivariate data, it can be used for univariate data using Euclidean distance (Anderson, 2017). Its advantage is that it properly handles asymmetrical and unbalanced designs, like this one. Additionally, as residuals were in general not normally distributed, the ANOVA using the traditional tabulated *F* values would be inappropriate. Therefore, the probability associated to each empirical *F*‐ratio was estimated using 9,999 permutations of the residuals under the reduced null model. To verify the assumptions for a correct interpretation of each ANOVA, graphical and quantitative analyses were performed (Figure S1). Also, the Levene's test for homogeneity of variances was used to assess the equality of variances when significant interactions were found in the ANOVA. When main effects or interactions effects were statistically significant, pairwise *t* tests based on permutation were applied. A probability level of 5% was used to test the significance of effects.

The temporal and spatial changes of the environmental variables were evaluated by applying the same multifactorial ANOVA model used for N_2_O and CH_4_ emissions to each variable. For the first significant interaction that included degradation level, status or microtopography, a paired *t* test with combined variances was applied after weighting by *n* and with probability correction for multiple comparisons using the Holm procedure (Holm, 1979). For each of the paired comparisons, probability values were obtained after the Holm adjustment. Soil properties and inorganic N were compared between sites within a microtopography, and between microtopographies within a site, using nonparametric tests as their residuals were not normally distributed according to the Shapiro–Wilks test.

Relationships between the fluxes and environmental variables were examined using monthly averages, annual averages and site‐scale annual values. Relationships with monthly averages were investigated site specifically at the microscale and by aggregating palm status and/or microtopography. They were also investigated across sites using classes for the independent variables (e.g., 10% intervals for the WFPS). Relationships with annual averages were tested across sites considering palm status and microtopography. Detection of emission hotspots was performed using boxplots analysis. A chamber qualified as a hotspot when it displayed over the monitored period at least three values higher than three times the interquartile range from the upper edge of the 50% percentile (i.e., extreme flux values; van den Heuvel et al., 2009).

The Gaussian error propagation method was used for propagating uncertainties of monthly averages and computing standard error of annual cumulative rates. Site‐scale annual cumulative or average values ± standard error which did not overlap were considered significantly different between sites/years. Results are presented as mean or cumulative values ± standard error. Statistical analyses were performed with the statistical software packages R (Core Team R, 2017) and PRIMER v7 (Anderson, 2017).

## 3 RESULTS

### 3.1 Soil properties and environmental variables

Edaphic properties were homogeneous among sites and microtopographies (Table S1). Bulk densities were low, and in hollows they were higher as degradation increased (from 0.08 to 0.11 g d.m./cm^3^; *p* = .0003). Soil macronutrients differed between sites only in hollows, with higher levels of Ca at the hDeg site than at the mDeg site and a higher concentration of Mn at the hDeg site than at the Intact site.

Over the whole measurement period, inorganic N pools were dominated by NH_4_^+^ (>380 mg N/kg d.m.) while NO_3_^−^ contents were quite low (<4 mg N/kg d.m.; Table 2). The absolute value of net mineralization rate (>18 mg N kg^−1^ d.m. day^−1^) was also much higher than net nitrification rate (<1 mg N kg^−1^ d.m. day^−1^). Soil NH_4_^+^ content was lower in hollows than in hummocks at the hDeg site (*p* < .0001). Likewise, net mineralization was lower in hollows than in hummocks at the Intact site (*p* < .0001), and in general (*p* = .05) corresponding to higher soil moisture levels (*p* < .0001). NH_4_^+^ content was lower at the hDeg site than at the other sites while net mineralization increased with degradation (*p* < .0001 for both). Soil mineral N content and dynamics did not follow a consistent temporal pattern but in April 2017 overall NH_4_^+^ contents, net mineralization and net nitrification were at their maximum, while NO_3_^−^ contents were at their minimum (*p* < .0001; Figure S2d,e). Monthly NH_4_^+^ content decreased when soil respiration raised in hummocks at the hDeg site (*R* ^2^ = .55, *p* = .03) and increased when the water level went up in hollows at the hDeg site (*R* ^2^ = .57, *p* = .03). Monthly net nitrification rate decreased when the soil WFPS rose in hollows at the mDeg site (*R* ^2^ = .55, *p* = .03).

**TABLE 2:** Monthly mean ± *SE* (*n* = 72) soil gravimetric moisture (*m*_G_), NH_4_^+^ and NO_3_^−^ contents, net mineralization rate (Net Min) and net nitrification rate (Net Nit) around live palms at the Intact, moderately (mDeg) and heavily (hDeg) degraded sites according to microtopography

Site	*m*_G_ (%)	NH_4_^+^ (mg N kg^−1^ d.m.)	NO_3_^−^ (mg N kg^−1^ d.m.)	Net Min (mg N kg^−1^ d.m. day^−1^)	Net Nit (mg N kg^−1^ d.m. day^−1^)
Intact hummock	478.8 ± 16.0^a1^	806.9 ± 56.7^2^	3.8 ± 0.7	−18.8 ± 10.6^b1^	0.0 ± 0.1
Intact hollow	1,032.4 ± 44.0^b2^	1,031.5 ± 103.5^2^	2.6 ± 0.6	−63.4 ± 9.1^a1^	0.1 ± 0.1
mDeg hummock	643.1 ± 18.8^a2^	907.9 ± 61.7^2^	4.0 ± 1.0	87.4 ± 28.3^2^	0.4 ± 0.2
mDeg hollow	980.3 ± 19.1^b12^	930.7 ± 68.1^2^	3.0 ± 0.8	30.9 ± 15.8^2^	0.2 ± 0.1
hDeg hummock	640.3 ± 35.6^a2^	571.9 ± 57.0^b1^	2.2 ± 0.6	80.4 ± 28.1^2^	1.1 ± 0.4
hDeg hollow	856.0 ± 12.5^b1^	389.5 ± 53.9^a1^	2.7 ± 0.7	52.4 ± 20.2^3^	0.4 ± 0.3

Note

Lowercase letters indicate significant differences between microtopographies within a site. Numbers indicate significant differences between sites within a microtopography. No letters or numbers are displayed in the absence of a significant difference.

**FIGURE 2:**
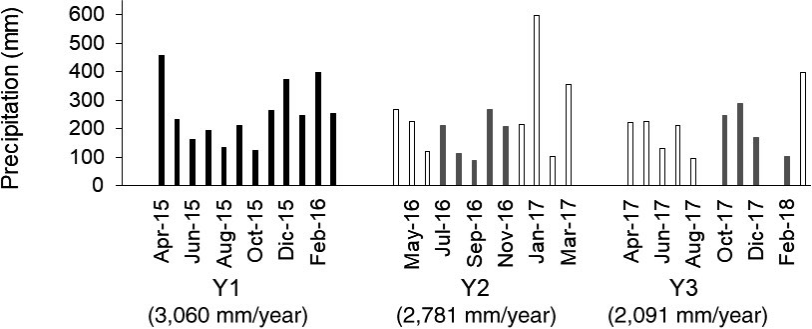
Monthly precipitation and annual precipitation per year of the monitoring period. Black bars show months with El Niño conditions. Gray bars show precipitations in months with weak La Niña conditions. White bars show El Niño/La Niña neutral months. Y, year

Monthly and annual precipitation during the measurement period are presented in Figure 2. The first year was under the influence of El Niño while 5 months of each the second and third year had weak La Niña conditions. Annual precipitation decreased from year 1 through year 3, with a rate in year 1 within the long‐term average of 3,087 mm/year. Following long‐term patterns, the highest monthly precipitations (>400 mm) occurred during the months from January to April and the lowest (<200 mm) between June and September, with no apparent connection to El Niño and La Niña events. High precipitation months included large daily rainfalls between 50 and 120 mm. Months with six consecutive days or more without precipitation were common during the period June to September, independently of the El Niño or La Niña events.

**FIGURE 3:**
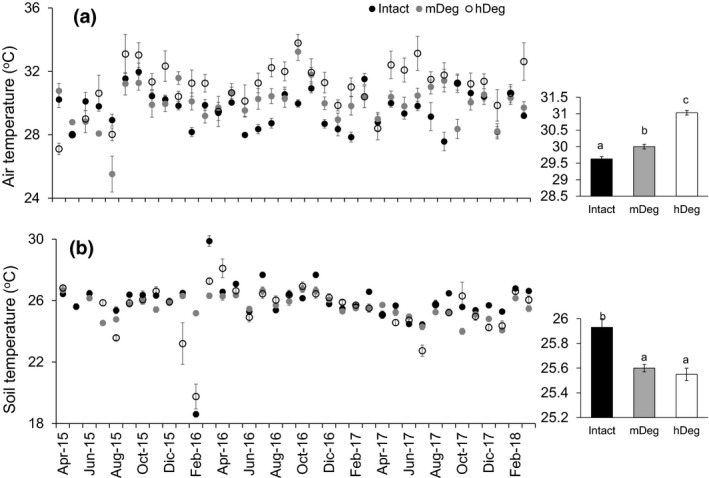
Monthly mean air (a) and soil (b) temperatures at the Intact, moderately (mDeg) and heavily (hDeg) degraded sites. Error bars are *SE*. Left charts display means per month (*n* = 18 at the Intact site, *n* = 36 at the degraded sites); right charts display 3‐year means (*n* = 648, 1,297 and 1,296, respectively at the Intact, mDeg and hDeg). Letters indicate significant differences in means between sites

The air temperature varied over time (*F*_66,2,708_ = 9.38, *p* < .001) between 28 and 32°C, 26 and 33°C and 27 and 34°C at the Intact site, mDeg site and hDeg site, respectively, with no apparent link to El Niño or La Niña events (Figure 3a left chart). Air temperature averaged over the 3 years indicated a warmer microclimate with increasing degradation (Figure 3a right chart). The soil temperature was homogeneous among microtopographies except the Intact site where hummocks (26.1 ± 0.1) were warmer than hollows (25.8 ± 0.1). Soil temperature varied over time (*F*_2,3,530_ = 32.26, *p* < .0001), with monthly means fluctuating between 19 and 30°C at the Intact site, 24–27°C at the mDeg site and 20–28°C at the hDeg site (Figure 3b left chart). Soil temperature over the 3 years was higher at the Intact site than at the degraded sites (Figure 3b right chart).

**FIGURE 4:**
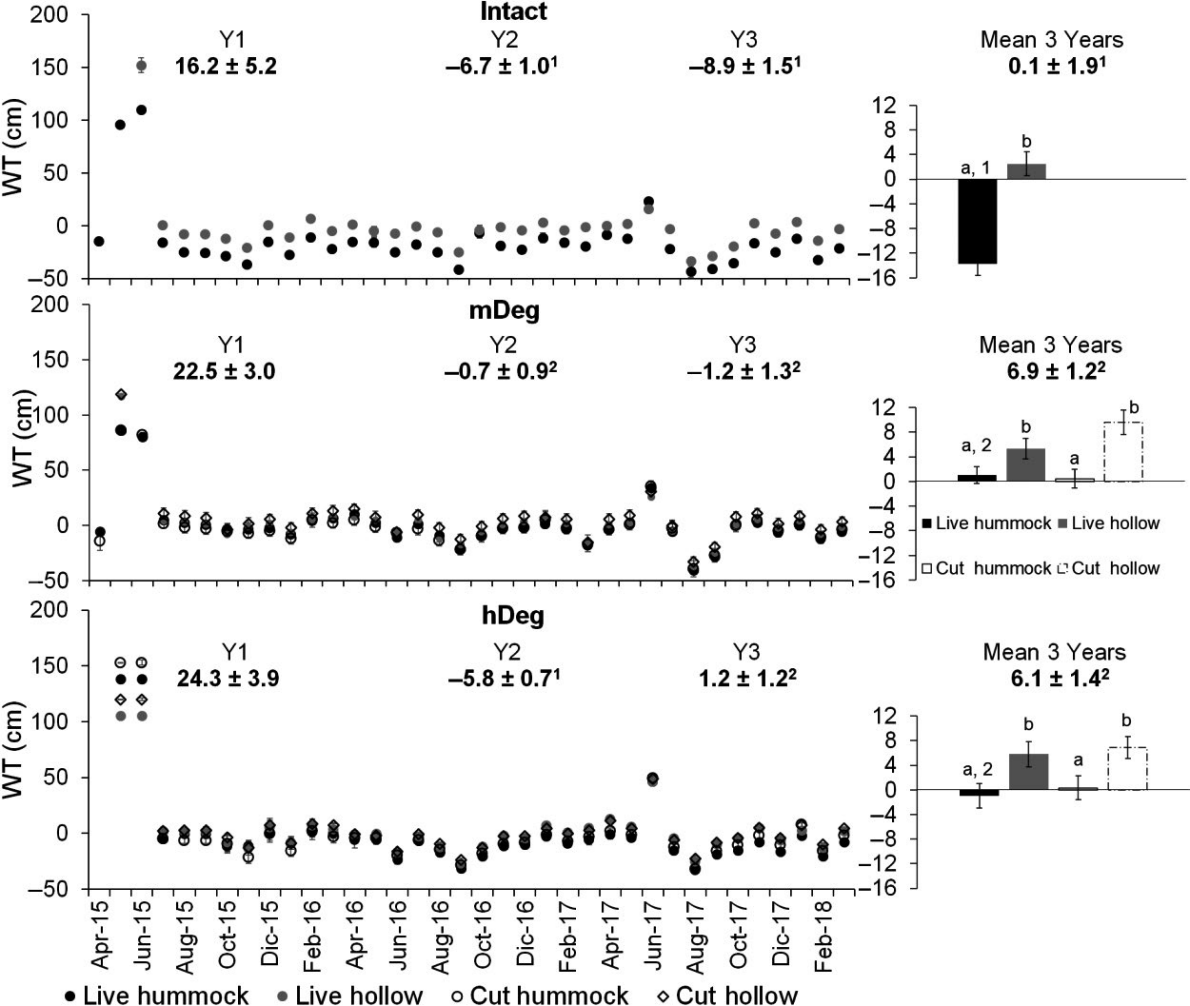
Monthly and annual mean water table level in hummocks and hollows around live and cut palms at the Intact, moderately (mDeg) and heavily (hDeg) degraded sites. Error bars are *SE*. Left charts displays means per month (*n* = 9 per palm status by spatial position at each site) and site‐scale annual means per year. Right charts display 3‐year means per palm status by spatial position (*n* = 308–324) and at site scale. Letters indicate significant differences in means between microtopographies within a site and palm status. Numbers indicate significant differences in means between sites

The WT was higher in hollows than in hummocks at all sites and for both live and cut palms (Figure 4 right chart). It was lower at the Intact site than at the degraded sites in hummocks, and at the site scale over the 3 years (Figure 4 right chart). Site‐scale WT level was similar among sites in the first year, it was lower at the Intact and hDeg sites than at the mDeg site in year 2, and it was lower at the Intact site than at the degraded sites in year 3 (Figure 4 left chart). The WT varied markedly over time (*p* < .0001). In May and June 2015, the sites flooded, and the WT rose to 80–150 cm above the soil surface. These months were affected by the El Niño event and were preceded by 2 months of very high rainfall, between 450 and 530 mm (Figure 2). Another flooding event affected all sites in June 2017, when the WT reached levels between 16 and 46 cm above the soil surface. Neither that month nor the previous month had the rainfall been higher than usual (Figure 2).

**FIGURE 5:**
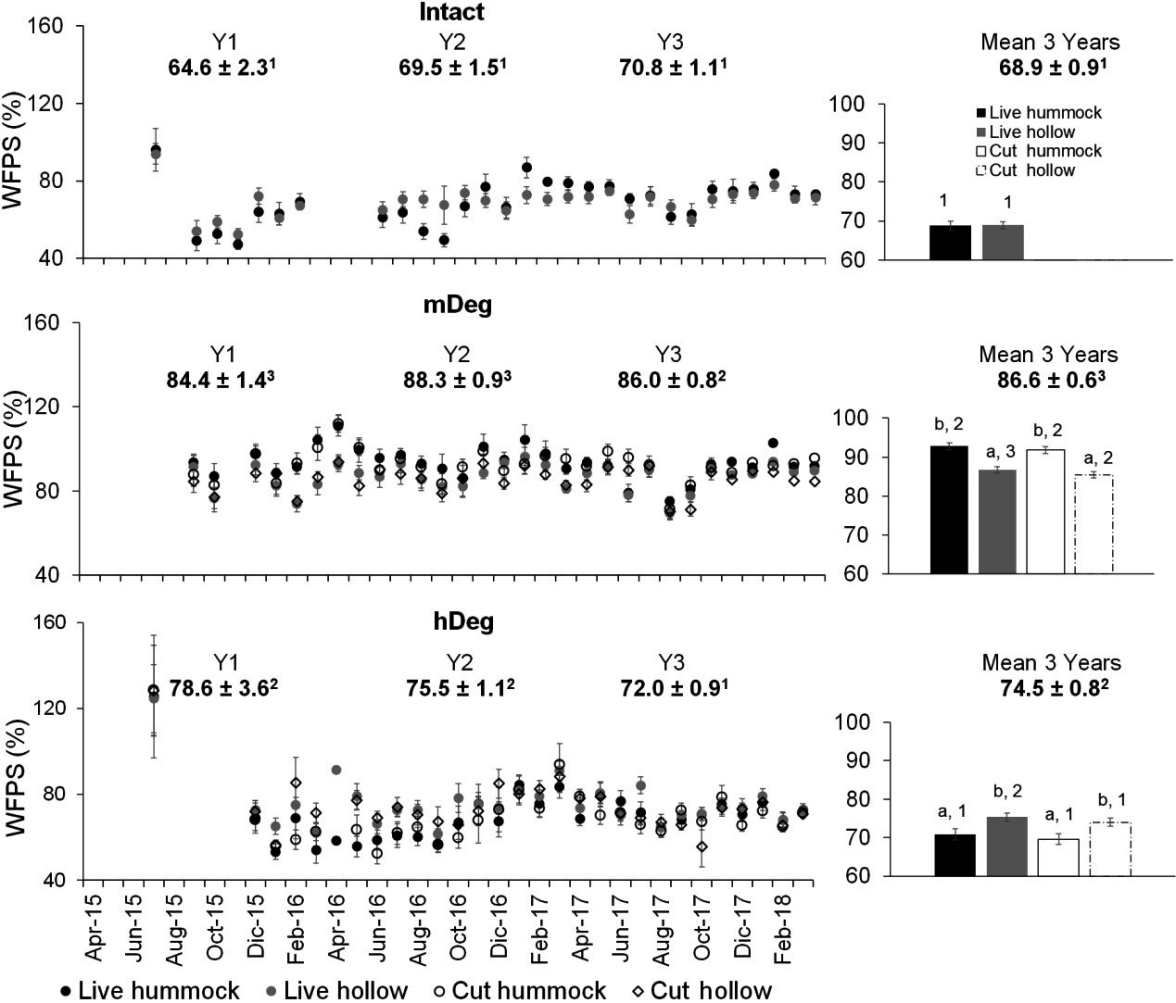
Monthly and annual mean water‐filled pore space in hummocks and hollows around live and cut palms at the Intact, moderately (mDeg) and heavily (hDeg) degraded sites. Error bars are *SE*. Left charts displays means per month (*n* = 9 per palm status by spatial position at each site) and site‐scale annual means per year. Right charts display 3‐year means per palm status by spatial position (*n* = 241–260) and at site scale. Letters indicate significant differences in means between microtopographies within a site and palm status. Numbers indicate significant differences in means between sites

The soil WFPS was similar among microtopographies at the Intact site (Figure 5 right chart). It was higher in hummocks than in hollows at the mDeg site for both live and cut palms, while the opposite occurred at the hDeg site. Over the 3 years, the WFPS was higher at the mDeg site than at the Intact and hDeg sites, in hummocks and hollows and for both live and cut palms (Figure 5 right chart). Site‐scale annual WFPS followed the order Intact < hDeg < mDeg over the 3 years and in years 1 and 2 while in the third year it was lower at Intact and hDeg sites than at the mDeg site. The WFPS fluctuated over time (*p* < .0001), with high values in July 2015 following the May–June flooding.

### 3.2 Soil N_2_O fluxes

**FIGURE 6:**
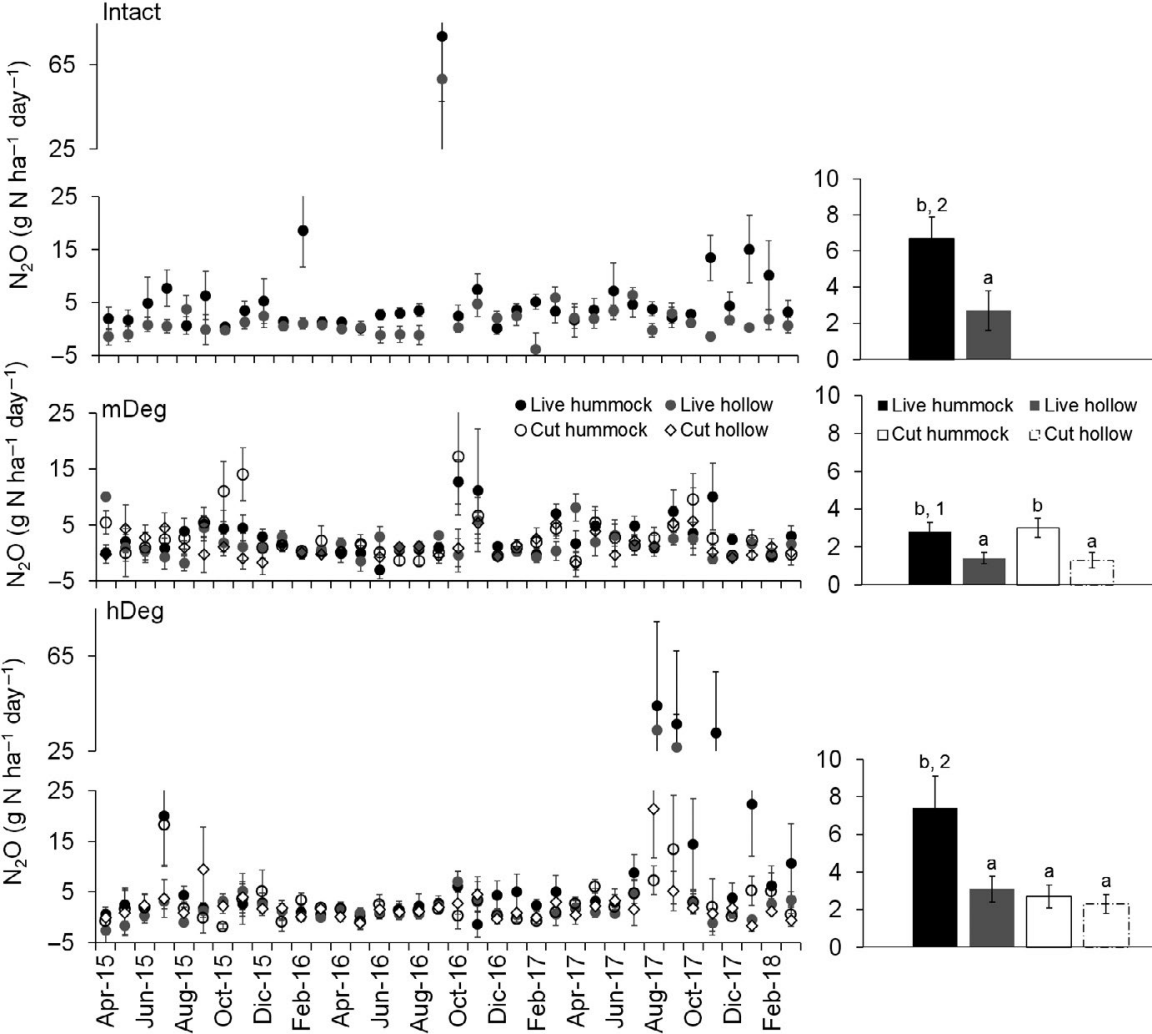
Monthly mean N_2_O emissions in hummocks and hollows around live and cut palms at the Intact, moderately (mDeg) and heavily (hDeg) degraded sites. Error bars are *SE*. Left charts displays means per month (*n* = 9 per palm status by spatial position); right charts display 3‐year means (*n* = 316–320 per palm status by spatial position). In left charts, note different scale between top (Intact), bottom (hDeg) and middle (mDeg) panels. Letters indicate significant differences in means between microtopographies within a site and palm status. Numbers indicate significant differences in means between sites within a microtopography and palm status

Mean monthly N_2_O emissions were higher in hummocks than in hollows at all sites around live palms and at the mDeg site around cut palms (Figure 6 right charts). Emissions were higher at the Intact and hDeg sites than at the mDeg site in hummocks supporting live palms (*F*_1,3,432_ = 10.60; *p* = .0025).

Measured flux values were 84% within the [−6.8; 6.8] g N ha^−1^ day^−1^ detection limit band. Monthly N_2_O fluxes fluctuated over a wide range, from small uptakes (−4 g N ha^−1^ day^−1^) to high emissions (79 g N ha^−1^ day^−1^; Figure 6 left charts). The range was the widest at the Intact site followed by the hDeg and mDeg sites. Uptake was frequent over the measurement period, particularly in hollows. There was also a tendency toward high emissions in months with less precipitation when the WT was below the soil surface, notably in September 2016 (90 mm) at the Intact site and in August 2017 at the hDeg site (95 mm). These high emissions contributed as much as 84% to year 2 emissions at the Intact site and 64% to year 3 emissions at the hDeg site. One chamber was identified as an emission hotspot. It was located on a hummock supporting a live palm at the hDeg site and presented extreme emission rates above 200 g N ha^−1^ day^−1^ in August, September and November 2017. Temporal dynamics of N_2_O fluxes and their magnitude differed between the sites (*F*_77,3,432_ = 5.70; *p* < .001), with alternating monthly increases and decreases.

**FIGURE 7:**
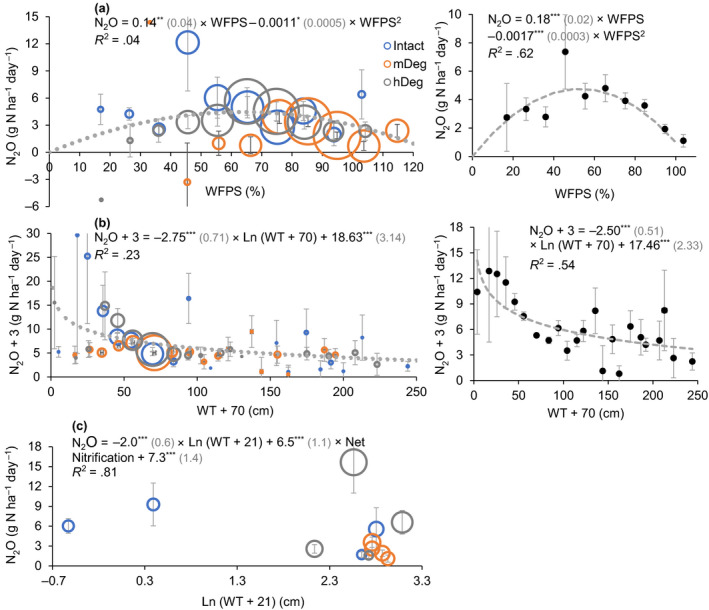
Relationship between monthly average (a, b) or annual average (c) N_2_O emissions and the water‐filled pore space (WFPS), the water table level (WT) and net nitrification rate. (a, b) present monthly emissions averaged within the respective WFPS (10% interval) and WT (10 cm interval) classes, disaggregated by site in left charts and all sites combined in right charts. In (c), annual averages were disaggregated by microtopography per site (excludes year 1 when mineral N data were not monitored). Black dots or bubbles and bars present average values and *SE*; dashed gray lines show the models. The models are presented with coefficients (*SE*) and their level of significance (*p < .05, **p < .01, ***p < .001). The size of the bubbles is relative to the sample size in (a) and (b); it is relative to net nitrification rate in (c) (range −0.3 to 1.8 mg N kg^−1^ day^−1^)

Monthly average N_2_O fluxes were mainly driven by variations in mineral nitrogen content and dynamics at the Intact site (Table 3, Equations 1–6). At the degraded sites, average fluxes were essentially linked to soil respiration and soil WFPS (Equations 7–15). Across sites, the relationship between monthly average N_2_O fluxes and the WFPS followed a polynomial trend with highest emissions around 55% WFPS (Figure 7a). Monthly soil N_2_O emissions also decreased logarithmically as the WT rose (Figure 7b). The power of these relationships increased substantially when N_2_O emissions were averaged among sites (Figure 7a,b right panels). Annual average emissions decreased with combined increased WT and decreased net nitrification rate (Figure 7c). This relationship showed a high explanatory power (*R* ^2^ = .81).

**TABLE 3:** Relationships between monthly average N_2_O fluxes and environmental parameters at the Intact, moderately (mDeg) and heavily (hDeg) degraded sites according to palm status (live, cut) and microtopography (hummock, hollow). Site‐specific relationships aggregated by palm status and/or microtopography are also presented

Model	*R* ^2^	*n*	Eq.
Ln (N_2_O + 4)_Intact live hummock_ = −0.01** (0.00) × Net Min + 1.84*** (0.10)	.71	8	(1)
Ln (N_2_O + 4)_Intact live hummock_ = 0.12* (0.04) × NO_3_^–^ + 1.64*** (0.16)	.64	8	(2)
N_2_O_Intact live hollow_ = 0.26* (0.07) × NO_3_^–^ − 1.4* (0.46) × Soil T + 37.2* (11.62)	.88	8	(3)
N_2_O _Intact live hollow_ = 0.33* (0.10) × NO_3_^–^ + 1.3* (0.51)	.66	8	(4)
Ln (N_2_O + 4)_Intact live_ = 0.07** (0.02) × NO_3_^–^ + 1.72*** (0.09)	.46	16	(5)
Ln (N_2_O + 4)_Intact live_ = − 0.38* (0.14) × Net Nit + 1.94*** (0.08)	.33	16	(6)
Ln (N_2_O + 4)_mDeg live hollow_ = 0.05** (0.02) × CO_2_ + 1.45*** (0.10)	.22	40	(7)
Ln (N_2_O + 4)_mDeg cut hummock_ = 0.07*** (0.02) × CO_2_ + 0.02* (0.01) × WT + 1.49*** (0.11)	.38	34	(8)
Ln (N_2_O + 4)_mDeg cut hummock_ = 0.04** (0.01) × CO_2_ + 1.56*** (0.11)	.25	40	(9)
Ln (N_2_O + 4)_mDeg cut hollow_ = 0.23*** (0.06) × Air T − 5.46** (1.79)	.32	34	(10)
Ln (N_2_O + 4)_hDeg live hollow_ = 0.05*** (0.01) × CO_2_ + 1.43*** (0.08)	.58	35	(11)
N_2_O_hDeg cut hummock_ = 0.16*** (0.04) × CO_2_ + 0.2*** (0.04) × WFPS − 14.40*** (3.36)	.48	35	(12)
Ln (N_2_O + 4)_hDeg cut hollow_ = 0.04*** (0.01) × CO_2_ + 1.42*** (0.08)	.37	35	(13)
N_2_O_hDeg cut hummock_ = 0.15** (0.05) × WFPS − 7.69* (3.44)	.24	35	(14)
Ln (N_2_O + 4)_hDeg_ = 0.03*** (0.00) × CO_2_ + 0.01** (0.00) × WFPS + 0.63* (0.29)	.27	40	(15)

Note

The models are presented with slope (*SE*), intercept (*SE*) and level of significance: *p < .05, **p < .01, ***p < .001. Soil N_2_O fluxes are expressed in g N ha^−1^ day^–1^, air (Air T) and soil (Soil T) temperatures are in °C, water table level (WT) is in cm, soil water‐filled pore space (WFPS) is in %, soil NO_3_^−^ content is in mg N/kg d.m., net mineralization (Net Min) and net nitrification (Net Nit) rates are in mg N kg^−1^ d.m. day^–1^ and soil respiration (CO_2_) is in kg C ha^–1^ day^–1^.

Site‐scale annual N_2_O emissions along the degradation gradient ranged from 0.5 ± 0.1 to 2.6 ± 0.7 kg N ha^−1^ year^−1^ (Table 4). At the Intact site, annual emissions were the highest in year 2, the lowest in year 1. At the mDeg site, N_2_O emissions remained steady over the years. At the hDeg site, emissions were the highest in year 3. These trends seemed linked to yearly fluctuations of the WFPS at the sites (Figure 7a) with most levels confined around 90% at the mDeg site (big bubbles in Figure 7a left panel) while at the Intact and hDeg sites the WFPS oscillated around a wide range of values. Site‐scale annual emissions were similar among sites in year 1 when the water level was high (>6 cm above ground). They were higher at the intact site than at the degraded sites in year 2, and followed the order hDeg > Intact > mDeg in year 3. Site‐scale annual emissions averaged over the 3 years were lower at the mDeg site than at the other two sites. As mentioned above, these differences seemed driven by inherent WFPS variations at the sites rather than to degradation.

**TABLE 4:** Cumulative annual and mean cumulative annual ± *SE* of N_2_O emissions (kg N ha^−1^ year^−1^) at the sites of the degradation gradient according to palm status (live, cut) and microtopography (hummock, hollow). Site‐scale annual values were computed using relative proportions from Table 1

Site	Annual Y1	Annual Y2	Annual Y3	Mean annual
Intact live hummock	1.6 ± 0.3	3.7 ± 0.8	2.3 ± 0.3	2.5 ± 0.6
Intact live hollow	0.3 ± 0.1	2.4 ± 0.9	0.6 ± 0.1	1.1 ± 0.6
Intact site‐scale	0.5 ± 0.1^α^	2.6 ± 0.7^2γ^	0.9 ± 0.1^2β^	**1.3 ± 0.6** ^2^
mDeg live hummock	0.8 ± 0.1	1.0 ± 0.2	1.4 ± 0.2	1.1 ± 0.2
mDeg live hollow	0.5 ± 0.1	0.4 ± 0.1	0.6 ± 0.1	0.5 ± 0.1
mDeg cut hummock	1.5 ± 0.2	1.0 ± 0.3	0.9 ± 0.2	1.1 ± 0.2
mDeg cut hollow	0.3 ± 0.2	0.6 ± 0.1	0.5 ± 0.2	0.5 ± 0.1
mDeg site‐scale	0.5 ± 0.1	0.5 ± 0.1^1^	0.6 ± 0.1^1^	**0.5 ± 0.1** ^1^
hDeg live hummock	1.3 ± 0.2	1.0 ± 0.2	5.8 ± 1.2	2.7 ± 1.5
hDeg live hollow	0.5 ± 0.1	0.6 ± 0.1	2.3 ± 0.4	1.1 ± 0.6
hDeg cut hummock	1.0 ± 0.3	0.3 ± 0.1	1.5 ± 0.3	1.1 ± 0.3
hDeg cut hollow	0.9 ± 0.3	0.5 ± 0.1	1.2 ± 0.3	0.9 ± 0.2
hDeg site‐scale	0.7 ± 0.1^α^	0.5 ± 0.1^1α^	1.9 ± 0.2^3β^	**1.1 ± 0.4** ^2^

Note

Values is bold highlight the site‐scale 3 years average at each site. Y1 = year 1 from April 2015 to March 2016; Y2 = year 2 from April 2016 to March 2017; Y3 = year 3 from April 2017 to March 2018; mDeg = moderately degraded; hDeg = heavily degraded. Cumulative annual emissions per palm status and microtopography were computed from 100 to 108 samples per site. *N* = 3 for mean cumulative annual emissions over 3 years. Greek letters indicate significant differences in site‐scale cumulative annual rate between years within a site. Number indicate significant differences between sites in site‐scale cumulative annual rate. No letters or numbers are displayed in the absence of a significant difference.

### 3.3 Soil CH_4_ fluxes

**FIGURE 8:**
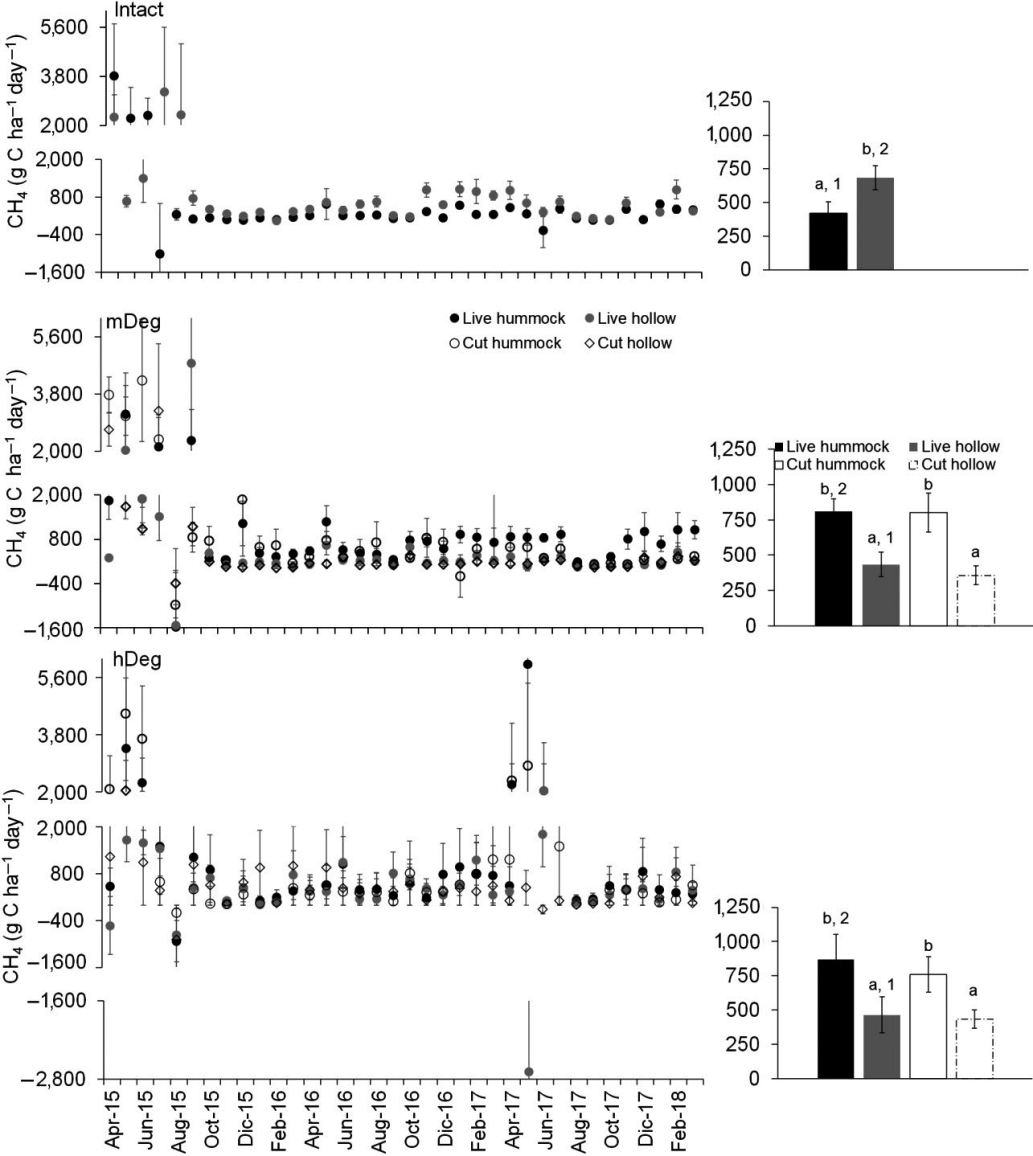
Monthly mean CH_4_ emissions in hummocks and hollows around live and cut palms at the Intact, moderately (mDeg) and heavily (hDeg) degraded sites. Error bars are *SE*. Left charts displays means per month (*n* = 9 per palm status by spatial position); right charts display 3‐year means (*n* = 312–318 per palm status by spatial position). In left charts, letters indicate significant differences in means between microtopographies within a site and palm status. Numbers indicate significant differences in means between sites within a microtopography and palm status

Mean monthly CH_4_ emissions were higher in hummocks than in hollows at both degraded sites around live and cut palms, while it was the opposite at the Intact site (Figure 8 right charts). The emissions were lower at the Intact site than at the degraded sites in hummocks supporting live palms. Conversely, emissions were higher at the Intact site than at the degraded sites in hollows surrounding live palms.

Measured flux values were 4% within the [−2; 2] g C ha^−1^ day^−1^ detection limit band. These low fluxes occurred when the WT was on average low. Monthly fluxes fluctuated over a wide range, from large uptake (−2,685 g C ha^−1^ day^−1^) to high emissions (6,021 g C ha^−1^ day^−1^; Figure 8 left charts). The range was the widest at the hDeg site followed by the mDeg site and the Intact site. The highest emissions occurred in hummocks while the highest uptakes occurred in hollows. Uptake was infrequent, occurring in 9 of the 48 months under a wide range of monthly precipitation (131–598 mm) and WFPS levels (58%–93%), independent of sites, palm status and microtopography. One chamber was identified as a hotspot. It was located in a hollow around a live palm at the Intact site and acted either as a strong source (July and August 2015, months affected by El Niño) or a strong sink (September 2014 and March 2015). Temporal dynamics of CH_4_ fluxes and their magnitude differed between sites (*F*_77,3,405_ = 1.73; *p* = .032), but fluxes were consistently large across sites following the May–June 2015 flooding.

**FIGURE 9:**
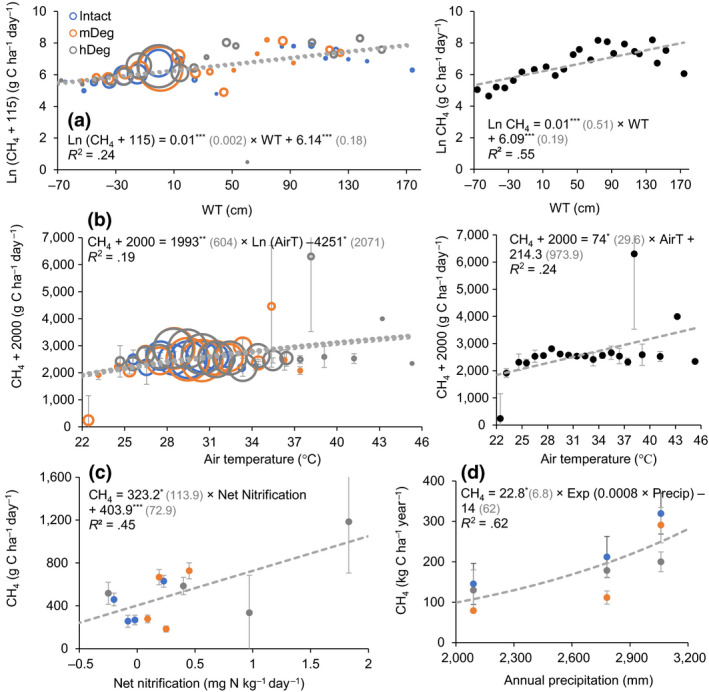
Relationship between monthly average (a, b), annual average (c) or site‐scale annual (d) CH_4_ fluxes and the water table level (WT), air temperature, net nitrification rate and annual precipitation. (a, b) present monthly emissions averaged within the respective WT (10 cm interval) and temperature (1°C interval) classes, disaggregated by site in left charts and all sites combined in right charts. In (c), annual averages were disaggregated by microtopography per site (excludes year 1 when mineral N data were not monitored). Black dots or bubbles present average values and *SE*; dashed gray lines show the models. In (a), as the *SE* of log‐transformed data can be misleading *SE* are not displayed. The models are presented with coefficients (*SE*) and their level of significance (*p < .05, **p < .01, ***p < .001). The size of the bubbles in (a) and (b) is relative to the sample size

Monthly average CH_4_ fluxes were linked to the WT at all sites (Table 5, Equations 16, 17, 21, 24–26, 28 and 30). At the degraded sites, average fluxes were also driven by mineral N content and dynamic, air temperature, soil WFPS (Equations 18–20, 23 and 29) and soil respiration (Equations 22 and 27). Across sites, monthly soil CH_4_ emissions increased exponentially as the WT rose (Figure 9a) and increased linearly as air temperature went higher (Figure 9b). The power of these relationships improved when CH_4_ emissions were averaged among sites (Figure 9a,b right panels), especially the relationship with the WT. Annual average emissions were positively related to net nitrification rate (Figure 9c). Finally, site‐scale annual emissions responded exponentially to annual precipitation across sites (Figure 9d).

**TABLE 5:** Relationships between monthly average CH_4_ fluxes and environmental parameters at the Intact, moderately (mDeg) and heavily (hDeg) degraded sites according to palm status (live, cut) and microtopography (hummock, hollow)

Model	*R* ^2^	*n*	Eq.
CH_4 Intact live hummock_ = 14.24*** (3.27) × WT + 614.15*** (100.46)	.30	47	(16)
CH_4 mDeg live hummock_ = 18.48*** (4.59) × WT + 786.12*** (101.30)	.30	40	(17)
CH_4 mDeg live hollow_ = 105.45*** (10.35) × Air T + 0.13* (0.04) × NH_4_^+^ − 2,994.02*** (314.18)	.96	8	(18)
CH_4 mDeg live hollow_ = 103.23*** (12.32) × Air T + 0.48* (0.18) × Net Min − 2,823.16** (364.24)	.94	8	(19)
CH_4 mDeg live hollow_ = −47.88** (11.3) × WFPS + 25.06* (8.14) × NO_3_^–^ + 4,511.85** (1,015.8)	.80	8	(20)
CH_4 mDeg cut hummock_ = 23.14*** (7.12) × WT + 767.13** (158.30)	.22	40	(21)
CH_4 mDeg live_ = 21.34* (10.54) × CO_2_ + 15.19*** (3.35) × WT + 388.58** (120.72)	.22	80	(22)
CH_4 hDeg live hummock_ = 137.74*** (12.52) × NO_3_^–^ + 478.91*** (52.58)	.95	8	(23)
CH_4 hDeg live hummock_ = 21.42*** (4.53) × WT + 934.33*** (135.22)	.35	44	(24)
CH_4 hDeg cut hummock_ = 27.71*** (3.36) × WT + 753.13*** (98.40)	.62	44	(25)
CH_4 hDeg cut hollow_ = 7.58*** (2.04) × WT + 379.60*** (63.89)	.25	44	(26)
CH_4 hDeg live_ = 18.18* (6.94) × CO_2_ + 16.02*** (3.06) × WT + 386.51* (146.69)	.25	88	(27)
CH_4 hDeg cut_ = 16.44*** (2.32) × WT + 538.68*** (70.53)	.37	88	(28)
CH_4 hDeg_ = 144.13*** (22.66) × NO_3_^–^ + 325.80** (93.22)	.74	16	(29)
CH_4 hDeg_ = 14.95*** (1.91) × WT + 616.09*** (58.22)	.26	176	(30)

Note

The models are presented with slope (*SE*), intercept (*SE*) and level of significance: *p < .05, **p < .01, ***p < .001. Soil CH_4_ fluxes are expressed in g C ha^−1^ day^−1^, air temperature (Air T) is in °C, water table level (WT) is in cm, soil water‐filled pore space (WFPS) is in %, soil NO_3_^−^ and NH_4_^+^ content are in mg N/kg d.m., net mineralization (Net Min) rate is in mg N kg^−1^ d.m. day^−1^, and soil respiration (CO_2_) is in kg C ha^−1^ day^−1^.

Site‐scale annual CH_4_ emissions along the degradation gradient ranged from 79.2 ± 5.5 to 319.4 ± 77.5 kg C ha^−1^ year^−1^ (Table 6) and were 1.5–4 times higher in the first year (El Niño) than in the third year (ENSO neutral; Figure 9d). They were higher at the Intact and mDeg sites than at the hDeg site in year 1, in the order Intact > hDeg > m Deg in year 2 and displayed no difference between sites in the last year. Over the 3 years, annual CH_4_ emissions were on average similar among sites.

**TABLE 6:** Cumulative annual and mean cumulative annual ± *SE* of CH_4_ emissions (kg C ha^−1^ year^−1^) at the sites of the degradation gradient according to palm status (live, cut) and microtopography (hummock, hollow). Site‐scale annual values were computed using relative proportions from Table 1

Site	Annual Y1	Annual Y2	Annual Y3	Mean annual
Intact live hummock	180.9 ± 47.2	97.3 ± 12.2	88.0 ± 14.2	122.1 ± 29.6
Intact live hollow	343.8 ± 90.9	232.2 ± 12.9	155.4 ± 13.4	243.8 ± 54.7
Intact site‐scale	319.4 ± 77.5^2γ^	212.0 ± 11.1^3β^	145.3 ± 11.5^α^	**225.6 ± 50.7**
mDeg live hummock	345.9 ± 57.1	243.9 ± 18.3	257.6 ± 19.8	282.5 ± 32.0
mDeg live hollow	290.5 ± 54.6	103.3 ± 9.0	63.8 ± 6.6	152.6 ± 69.9
mDeg cut hummock	438.3 ± 67.9	317.6 ± 84.9	97.9 ± 10.1	284.6 ± 99.6
mDeg cut hollow	244.6 ± 55.0	60.0 ± 5.6	59.7 ± 5.8	121.4 ± 61.6
mDeg site‐scale	290.9 ± 43.9^2γ^	111.5 ± 16.3^1β^	79.2 ± 5.5^α^	**160.5 ± 65.9**
hDeg live hummock	276.0 ± 50.2	221.7 ± 21.8	423.6 ± 121.4	307.1 ± 60.3
hDeg live hollow	178.9 ± 36.6	186.7 ± 27.3	142.5 ± 83.5	169.4 ± 13.6
hDeg cut hummock	292.0 ± 52.7	193.8 ± 29.5	289.2 ± 65.2	258.3 ± 32.3
hDeg cut hollow	218.5 ± 30.1	157.9 ± 21.8	82.8 ± 24.8	153.1 ± 39.2
hDeg site‐scale	199.9 ± 24.5^1^	178.5 ± 18.1^2^	129.8 ± 50.2	**169.4 ± 20.7**

Note

Values is bold highlight the site‐scale 3 years average at each site. Y1 = year 1 from April 2015 to March 2016; Y2 = year 2 from April 2016 to March 2017; Y3 = year 3 from April 2017 to March 2018; mDeg = moderately degraded; hDeg = heavily degraded. Cumulative annual emissions per palm status and microtopography were computed from 100 to 108 samples per site. *N* = 3 for mean cumulative annual emissions over 3 years. Greek letters indicate significant differences in site‐scale cumulative annual rate between years within a site. Numbers indicate significant differences between sites in site‐scale cumulative annual rate. No letters or numbers are displayed in the absence of a significant difference.

## 4 DISCUSSION

### 4.1 Soil properties, inorganic N contents and dynamics and moisture fluctuations along the degradation gradient

The soil in the study area was a mosaic of microtopographic variation formed by hummocks and hollows. At all three sites, hollows represented more than 80% of the total surface area (van Lent et al., 2019; Table 1), contrary to what has been reported for Southeast Asia, where hollows form around 50%–60% of the peat surface (Hergoualc’h, Hendry, et al., 2017; Jauhiainen et al., 2005; Swails et al., 2019). Degradation induced a higher hollow‐to‐hummock proportion at the degraded sites (91%–96%) than at the Intact site (85%) as the result of both a lower density of live *M. flexuosa* palms and a smaller area of the hummocks likely due to combined fostered organic matter decomposition and reduced aboveground and belowground litter inputs (van Lent et al., in preparation).

The topsoil was homogeneous along the degradation gradient and among microtopographies, with the exception of bulk density, Ca and Mg in hollows. The higher bulk density with increased degradation may reflect degradation‐induced soil compaction. Soils displayed high concentrations of Ca and Mg, high percentage of base saturation and high phosphorus levels. Large concentrations of these nutrients were also recorded in other peatlands of the region, resulting in their classification as minerotrophic (Lähteenoja, Ruokolainen, Schulman, Alvarez, et al., 2009) and indicating sediments inputs during river flooding periods. NH_4_^+^ contents were remarkably high with values comparable to levels measured in a peat swamp forest in Sumatra (500–3,000 mg N/kg d.m.; Hartill, 2017). A high level of NH_4_^+^ and a dominance of NH_4_^+^ over NO_3_^−^ in the inorganic‐N pool (Table 2) is typical of damp peat soils (e.g. Oktarita et al., 2017; Swails et al., 2017) and reflects an inhibition of nitrification due to limitation in O_2_ supply. This inhibition is evidenced by the low net nitrification rates, especially at the Intact site. Nitrification can also be inhibited by polyphenolic compounds (Zeller et al., 2007) which have been found to be important in Southeast Asian peat swamp forests (Yule et al., 2016). The lower NH_4_^+^ content at the hDeg site compared to concentrations at the other sites may be due to enhanced immobilization “in situ”. Cheng et al. (2014) found that litter decomposition in acidic forest soils could induce a shift from net N mineralization to net N immobilization. Therefore, the faster litter decomposition rate and lower litterfall C/N ratio at the hDeg than at the other sites (van Lent et al., 2019, in preparation) could partly explain differences in NH_4_^+^ contents between sites. The higher “in vitro” net mineralization rate with increasing degradation was not linked to soil moisture and cannot be attributed to soil properties which were homogeneous among sites as mentioned previously. A potential explanation and avenue for further research would be an alteration of microbial communities prompted by changes in botanical composition associated with degradation (Girkin et al., 2019; Zhou et al., 2017).

The sites showed some differences in WT and soil moisture especially in hummocks which represent a small share (<15%) of site‐scale surface. At this microtopography, the lower WT level at the Intact site than at the degraded sites and the lower WFPS at the Intact and highly degraded sites than at the site of medium degradation were consistent over time. Hollows displayed a similar WT level among sites (Figure 4 right charts) and a WFPS in the order mDeg > hDeg > Intact on average (Figure 5 right charts) though site differences were inconsistent over time as reflected by yearly site‐scale results. The mDeg site showed lower WT and WFPS fluctuations than the other two sites which likely results from its slightly higher elevation and distance from the Itaya river (van Lent et al., 2019).

### 4.2 Spatiotemporal variations of soil N_2_O fluxes along the degradation gradient and their controls

The high spatiotemporal variability of N_2_O fluxes found along the degradation gradient concurs with results by, for example, Inubushi et al. (2003) or Melling et al. (2007) in tropical peatlands. Variability of fluxes has been attributed to several factors, but especially to variations in WT and soil inorganic N (Martikainen et al., 1993; Melling et al., 2007; Oktarita et al., 2017). Spatial variability at the microscale was characterized by a general trend toward higher N_2_O emissions from hummocks than from hollows (Figure 6 left charts). This trend is consistent with drier conditions (see WT in Figure 4), higher soil organic matter decomposition (van Lent et al., in preparation) and larger soil net N mineralization rates in hummocks than in hollows (Table 2). The infrequent, irregular but very large N_2_O emissions (with maximum as high as 333 g N ha^−1^ day^−1^) and the presence of one hotspot at the sites could not be clearly associated with the environmental variables. Such high flux pulses have also been reported for other tropical peat forests (Jauhiainen et al., 2012; Takakai et al., 2006) and have been attributed to a series of plant and soil factors that govern oxygen diffusion and the fate of inorganic N (Oktarita et al., 2017). Most fluxes of N_2_O (84%) were within the detection limit band; as also reported by several studies conducted in undrained peatlands (Jordan et al., 2016; Wilson et al., 2013, 2016). The [−6.8; 6.8] g N ha^−1^ day^−1^ detection limit band was much lower than the 11–88 g N ha^−1^ day^−1^ limit for the method implemented by Jordan et al. (2016) in Swedish peatlands and similar to that of 6.1 g N ha^−1^ day^−1^ for the research by Oktarita et al. (2017) in Indonesian peatlands.

Despite the large spatiotemporal variability described above, the ranges for the 3‐year averages along the degradation gradient (1.3–7.4 g N ha^−1^ day^−1^) or for annual site‐scale rates (0.5–2.6 kg N ha^−1^ year^−1^; Table 4) were rather narrow. The average rates are in the same order of magnitude as the annual means of 0.6 and 2.0 g N ha^−1^ day^−1^ reported by Jauhiainen et al. (2012) and Melling et al. (2007) for undrained peat swamp forests in Southeast Asia but 200 to 1,000‐fold higher than the very low mean (0.007 g N ha^−1^ day^−1^) reported for similar *M. flexuosa* palm swamp forests in the Peruvian Amazon (Teh et al., 2017). Interannual site‐scale emissions fall within the range of fluxes from forested peatlands in Southeast Asia (0.5–13.4 kg N ha^−1^ year^−1^) and are close to the average of 2.7 ± 1.9 kg N ha^−1^ year^−1^ reported for intact tropical peat swamp forests (Hergoualc'h & Verchot, 2014). On an annual basis, all sites were N_2_O sources. Soil uptake of atmospheric N_2_O was frequent at all sites, particularly in hollows (Figure 6 left charts) and was registered over a wide range of soil moisture. N_2_O uptake has been detected in Southeast Asian peatlands (e.g., Jauhiainen et al., 2012; Takakai et al., 2006) and is usually favored by low nitrate availability, high WFPS and more generally conditions impeding N_2_O diffusion in the soil (Chapuis‐Lardy et al., 2007). These conditions were fulfilled at the sites where the soil was often water‐logged and nitrate‐limited (Table 2).

N_2_O fluxes were on average lower at the mDeg site than at the other sites; however, this difference seems more closely related to spatial variation in soil moisture fluctuation along the forest complex than to degradation (Figure 7a). When WT levels were high in year 1 emissions were similar among sites (Table 4) while emission peaks in year 2 at the Intact site (September 2016) and in year 3 at the hDeg site (August 2017; Figure 6 left charts) seemingly induced by precipitation drops contributed highly to large annual fluxes at these sites (Table 4). Furthermore, comparison between fluxes around live and cut palms at the degraded sites denote no degradation impact (Figure 6 right charts). Notwithstanding the difference in drivers of fluxes between the Intact and the degraded sites points toward some impacts of degradation at the microscale. For instance, at the mDeg and hDeg sites, fluxes were controlled by soil respiration, a variable which increased with degradation (van Lent et al., 2019, in preparation). The positive response of N_2_O fluxes to soil respiration in hummocks and hollows supporting live and cut palms highlights a control of soil organic matter decomposition over denitrification in medium and high degraded conditions (Table 3, Equations 7, 9 and 11–13). On the other hand, soil net mineralization rate which diminished with degradation was not related to fluxes at the degraded sites (Table 3) while together soil NO_3_^−^ content it exerted a strong control on emissions at the Intact site. Emissions in hummocks and hollows increased as “in situ” NO_3_^−^ contents got higher (Table 3, Equations 2–4) but decreased as “in vitro” net nitrification shifted from small negative to small positive rates (Table 3, Equation 6). This suggests that processes others than denitrification such as, for example, nitrifier denitrification (Wrage et al., 2001) contributed to N_2_O production. The strong capacity of soil NO_3_^−^ content to predict N_2_O emissions is in agreement with findings by Pärn et al. (2018) across a wide range of N‐rich organic soils.

The main controls of N_2_O fluxes across sites were the WFPS and the WT alone or in combination with net nitrification rate (Figure 7). A maximum of emissions along the peat forest complex around 55% WFPS (Figure 7a) is slightly below the 60%–70% WFPS for highest N_2_O emissions reported by van Lent et al. (2015) for mineral soils in the tropics and similar to the 50% soil moisture for the peak of the bell‐shaped curve found by Pärn et al. (2018). This mid‐point WFPS corresponds to optimal oxygen conditions for N_2_O production; while denitrification produces gradually more nitric oxide as the WFPS goes down and reduces N_2_O into N_2_ as the WFPS goes up (Davidson et al., 2000). The logarithmic decrease in N_2_O emissions as the WT rose (Figure 7b) is similar to the response found for peatlands in Southeast Asia (Hergoualc'h & Verchot, 2014) and denotes decreased oxygen supply for N_2_O production as sites get flooded. The best fit biogeochemical model included WT level and net nitrification rate and explained 81% of annual flux variation over years and across sites and microtopographies (Figure 7c). This relationship coincides with the positive and negative correlations between N_2_O fluxes and, respectively, nitrification potential and WT found by Regina et al. (1996) in boreal peatlands. Our results demonstrate that both WFPS and WT levels exert a strong control over N_2_O fluxes; however, annual emissions along the forest complex did not follow a consistent trend according to El Niño/La Niña. Natural soil N_2_O emissions in the northern Amazon have been predicted to decrease/increase during periods of El Niño/La Niña (Saikawa et al., 2013) with observed N_2_O emission reductions from El Niño to La Niña in equatorial regions, strongly correlated with precipitation and soil moisture changes. The impact of future El Niño/La Niña events on N_2_O emissions from palm swamp peatlands in the northwestern Amazon will depend very much on how precipitation will influence WFPS and WT levels.

### 4.3 Spatiotemporal variations of soil CH_4_ emissions along the degradation gradient and their controls

Soil–atmosphere CH_4_ exchange results from complex interactions between several processes controlling production, consumption, transport and release of the gas, and the dominance of any particular process at one site may not occur elsewhere, causing high spatial variability (Bartlett & Harris, 1993). Since CH_4_ production prevails in waterlogged conditions, most studies on CH_4_ fluxes in peat swamp forests have focused on hollows disregarding fluxes from hummocks (Jauhiainen et al., 2005). We found lower emissions in hummocks than in hollows at the Intact site which is consistent with current mechanistic understanding of CH_4_ fluxes (Le Mer & Roger, 2001). The larger emissions in hummocks than in hollows at the degraded sites may have arisen from several factors. First, the WT gap between the two microtopographies was lower at the degraded sites (7 cm on average) than at the Intact site (16 cm). Second, plant‐mediated emissions via pneumatophores mainly located in hollows could have been lower at the degraded than at the Intact site since pneumatophores density decreased with degradation (from 6 to 0.3–2 pneumatophores dm^−2^ at the intact and degraded sites, respectively; van Lent et al., 2019). Finally, the more readily decomposable litterfall at the degraded sites than at the Intact site (as indicated by its lower C/N ratio; van Lent et al., 2019) may have prompted CH_4_ emissions. Plant residues are known to be a major substrate for methanogens (Le Mer & Roger, 2001) and microbial decomposition in wetlands often correlates with C quality indexes (Bridgham et al., 2013). Spatial variation was also exemplified by the presence of one hotspot in a hollow at the Intact site. The hotspot acted either as a strong source in the months following the El Niño‐induced flooding when litterfall rates peaked (van Lent et al., in preparation) or as a strong sink. High CH_4_ uptakes have also been reported in peatlands of Panama (up to 1,152 g C ha^−1^ day^−1^, Wright et al., 2011). The [−2; 2] g C ha^−1^ day^−1^ detection limit band of our method was similar to limits reported by Bartlett et al. (1988; 0.8 g C ha^−1^ day^−1^) for Amazonian floodplains and Smith and Lewis (1992; 1 g C ha^−1^ day^−1^) for temperate wetlands and in the low range of the 1.5–9 g C ha^−1^ day^−1^ limit evaluated by Christensen (1993) for Arctic tundra. Given the large magnitude of the fluxes at the sites, only 4% of them fell within the detection limit band.

Emissions of CH_4_ were extremely variable over time (Figure 8 left charts). The 3‐year average emission rates (from 357 to 870 g C ha^−1^ day^−1^, Figure 8 right chart) are comparable to averages measured by Griffis et al. (2020) by eddy covariance at ecosystem scale at the Intact site (548–658 g C ha^−1^ day^−1^) and reported by Teh et al. (2017) for *M. flexuosa* palm swamp peatlands in the Peruvian Amazon (255–534 g C ha^−1^ day^−1^) but lower than values recorded in forested peatlands of Panama (384–3,024 g C ha^−1^ day^−1^; Wright et al., 2013). Mean annual site‐scale emissions (161–226 kg C ha^−1^ year^−1^, Table 6) are about seven times higher than the average reported for intact peat swamp forests in Southeast Asia (29 kg C ha^−1^ year^−1^; Hergoualc’h & Verchot, 2014). High CH_4_ emissions in comparison with those of Southeast Asia could be associated with a higher quality of organic matter (Swails et al., 2017) containing lower levels of lignin (Hatano et al., 2016). They could also be related to higher WT at our sites (0.2–7 cm at the site scale) than on average in undrained peat swamp forests of Southeast Asia (−18 ± 6 cm; Hergoualc’h & Verchot, 2014). As noted by Sjögersten et al. (2014), the relatively low CH_4_ emissions in Southeast Asian peatlands do not seem representative of tropical wetlands globally.

Degradation did not affect site‐scale annual emissions (Table 6) but seem to have impacted microscale fluxes (Figure 8 right charts). In hummocks, the lower average emission rate at the Intact than at the degraded sites could be related to differences in WT level among sites (Figure 4 right charts) though CH_4_‐WT relationships at this spatial position explained only 36% of flux variation on average (Table 5, Equations 16, 17, 21, 24 and 25). As earlier mentioned, alteration of litterfall quality at the degraded sites could have stimulated hummock emissions; possibly explaining the strong CH_4_‐NO_3_^–^ relationship at the hDeg site (Table 5, Equation 23). In hollows where the WT was even among sites (Figure 4 right charts), emissions were higher at the Intact site than at the degraded sites which could be linked to weakening of plant‐mediated emissions following degradation. We also found strong relationships between air temperature, NH_4_^+^ content, net mineralization rate and CH_4_ fluxes at the mDeg site (Table 5, Equations 18 and 19). Regardless of microtopography, monthly CH_4_ fluxes as was the case for N_2_O fluxes were positively linked to soil respiration rate at both degraded sites but not at the Intact site (Table 5, Equations 22 and 27).

Across sites, the WT level was the dominant control of monthly CH_4_ emissions (Figure 9a). WT fluctuations influence aerobic and anaerobic decomposition by displacing, respectively, the oxic and anoxic layers where CH_4_ is oxidized and produced. The depression of CH_4_ oxidation in response to raised WT level has been documented in numerous studies (Bridgham et al., 2013). To a lower extent, monthly variations were also influenced by air temperature (Figure 9b). Temperature is recognized as an important control over methanogenesis while it affects less methanotrophy (Le Mer & Roger, 2001). Annual average emissions over years and across sites and microtopographies increased when net nitrification rate went higher (Figure 9c). Increased mineral N content is generally believed to have the potential to enhance CH_4_ emissions due to its inhibitory effect on methanotrophy; however, few studies have been conducted in natural wetlands (Bodelier & Laanbroek, 2004). Using a long‐term experiment in a boreal mire, Eriksson et al. (2010) demonstrated that N deposition simulated by adding ammonium nitrate caused increased CH_4_ production but did not affect CH_4_ oxidation. Finally, annual site‐scale CH_4_ emissions were positively related to precipitation (Figure 9d). This relationship suggests that climate change predictions of higher precipitations in the northwest Amazon (Malhi et al., 2008; Marengo et al., 2008) may imply higher CH_4_ emissions from these palm swamp peatlands.

## 5 CONCLUSION

Tropical peatlands are of global and regional importance but remain critically understudied especially outside of Southeast Asia. Very little is known about their state of conservation and their contribution to mitigate and/or exacerbate climate change. Our study showed that degradation of palm swamp peatlands in the Peruvian Amazon modifies soil–atmospheric exchanges of N_2_O and CH_4_ at the microscale but not significantly at the macroscale. Some of the main environmental drivers of emissions for both gases (soil WFPS, WT) were moisture‐related suggesting that future changes in rainfall patterns in the region may substantially alter N_2_O and CH_4_ emissions. Our study also provided evidence of the significant magnitude of the emissions, particularly for CH_4_; highlighting the critical need for their inclusion in biogeochemical modeling of peatlands.

## Supporting information

Supplementary Material

## Data Availability

Raw and summary replication data of soil N_2_O and CH_4_ fluxes and environmental variables are available at https://doi.org/10.17528/CIFOR/DATA.00243.
