# Supplementary material for: Spatial and temporal variability of soil N2O and CH4 fluxes along a degradation gradient in a palm swamp peat forest in the Peruvian Amazon
Source: Glob Chang Biol. 2020 Oct 9;26(12):7198–216. doi: 10.1111/gcb.15354 (PMC7756671; doi:10.1111/gcb.15354)
Supplement: Supplementary file 1 — Supplementary Material [file GCB-26-7198-s001.pdf]

SUPPLEMENTARY INFORMATION

Spatial and temporal variability of soil N_2O and CH_4 fluxes along a degradation gradient in a palm swamp peat forest in the Peruvian Amazon

Kristell Hergoualc'h[1], Nelda Dezzeo[1,2], Louis V. Verchot[3], Christopher Martius[4], Jeffrey van Lent[1,5], Jhon del Aguila-Pasquel[6], Mariela Lopez[1]

[1] Center for International Forestry Research (CIFOR), Lima, Peru

[2] Venezuelan Institute for Scientific Research (IVIC), Caracas, Venezuela

[3] Center for International Tropical Agriculture (CIAT), Cali, Colombia

[4] Center for International Forestry Research (CIFOR), Bonn, Germany

[5] Department for Soil Quality, Wageningen UR, Wageningen, The Netherlands

[6] Instituto de Investigaciones de la Amazonia Peruana (IIAP), Iquitos, Peru

Distribution of residuals of N_2O and CH_4 fluxes

Distribution of residuals was assessed using Q-Q plot, Violin plot and the Shapiro-Wilk test for normality, the Anscombe-Glynn test for Kurtosis and the D'Angostino test for symmetry. For both N_2O and CH_4, the Q-Q plots (Figure S1a, b) showed that the residuals deviated from a standardized normal distribution. The high density of points between the expected -2 and 2 quantiles suggested an almost symmetric distribution, but the very high frequency of extreme values (tails deviating from the expected line) indicated a strongly leptocurtic distribution. The violin plots (Figure S1c, d) showed that the data from the three sites exhibited a very similar distribution pattern. The Shapiro-Wilk test indicated that the residuals were not normal distributed ($W = 0.45248$, $p < 0.0001$ for N_2O; and $W = 0.49445$, $p < 0.0001$ for CH_4). Likewise, the D'Angostino symmetry test indicated that the residuals tended to be right-skewed (skewness = 9.903, $z = 63.777$, $p < 0.001$ for N_2O; and skewness = 4.5713, $z = 48.2510$, $p < 0.001$ for CH_4).

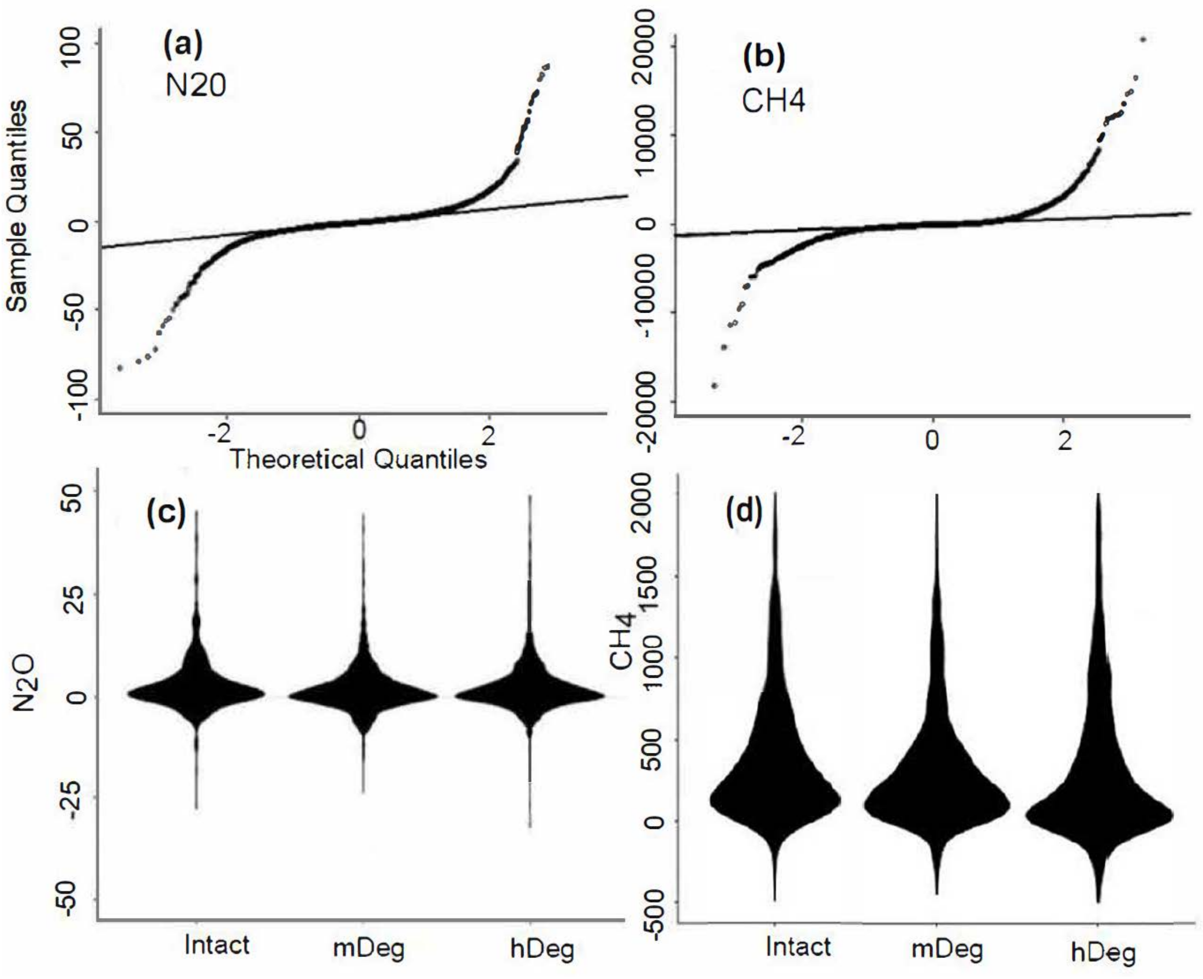

Figure S1: Normal Q-Q plots and Violin plots for N_2O (g N ha^{-1} d^{-1}) (panels (a), (c)) and CH_4 (g C ha^{-1} d^{-1}) (panels (b), (d)) for the Intact, moderately (mDeg) and heavily (hDeg) degraded sites.

Edaphic properties

Table S1: Soil characteristics (0–5 cm) at the Intact, moderately (mDeg) and heavily (hDeg) degraded sites according to microtopography (hummocks and hollows). Values are given as mean ± standard error (n = 3, except for bulk density (BD) for which n = 9).

	Intact		mDeg		hDeg	
	Hummocks	Hollows	Hummocks	Hollows	Hummocks	Hollows
BD	0.09 ± 0.01	0.08 ± 0.01[a]	0.10 ± 0.01	0.09 ± 0.00[b]	0.10 ± 0.01	0.11 ± 0.00[c]
N	2.5 ± 0.1	2.7 ± 0.3	2.5 ± 0.1	2.8 ± 0.1	2.5 ± 0.1	2.5 ± 0.1
C	44.9 ± 1.1	43.2 ± 3.2	43.2 ± 1.2	45.5 ± 0.5	35.4 ± 2.2	35.1 ± 2.3
C/N	17.9 ± 0.6	16.3 ± 0.4	17.8 ± 1.2	16.0 ± 0.1	14.4 ± 0.6	13.9 ± 0.3
Ca	3.9 ± 1.44	5.6 ± 0.6[ab]	5.0 ± 1.4	4.1 ± 0.4[a]	6.8 ± 0.5	7.4 ± 0.1[b]
K	0.5 ± 0.1	0.3 ± 0.1	0.5 ± 0.1	0.5 ± 0.1	0.8 ± 0.2	0.6 ± 0.2
Mg	0.7 ± 0.0	0.3 ± 0.0	0.4 ± 0.1	0.3 ± 0.0	0.5 ± 0.1	0.5 ± 0.1
Na	0.1 ± 0.0	0.1 ± 0.0	0.1 ± 0.0	0.1 ± 0.0	0.1 ± 0.11	0.1 ± 0.0
CEC	82.4 ± 2.8	68.9 ± 7.4	77.0 ± 6.4	70.1 ± 2.9	72.0 ± 7.2	68.3 ± 2.4
BS	31.8 ± 7.3	47.1 ± 7.3	39.1 ± 7.4	35.0 ± 2.4	57.9 ± 8.9	62.5 ± 2.7
P	273.7 ± 24.0	175.6 ± 50.7	272.5 ± 31.7	222.1 ± 76.8	76.1 ± 25.8	67.2 ± 19.5
Cu	0.2 ± 0.0	0.2 ± 0.0	0.2 ± 0.0	0.1 ± 0.0	0.2 ± 0.0	0.2 ± 0.0
Mn	136.6 ± 22.3	23.8 ± 3.6[a]	238.5 ± 74.1	52.1 ± 8.5[ab]	95.9 ± 2.8	84.5 ± 7.1[b]
Zn	10.0 ± 2.8	11.2 ± 0.5	9.9 ± 2.4	16.8 ± 1.1	9.0 ± 0.8	10.6 ± 2.2

Letters indicate a significant difference between sites within a spatial position ($p < 0.05$). No letters are displayed in the absence of a significant difference. BD = soil bulk density (g d.m. cm^{-3}); N = nitrogen (%), C = carbon (%); Ca = calcium, K = potassium, Mg = magnesium, Na = sodium (g kg^{-1} d.m.); CEC = cation exchange capacity ($cmol_c$ kg^{-1} d.m.); BS = base saturation (%); P = phosphorous, Cu = cupper, Mn = manganese, Zn = zinc (mg kg^{-1}).

Temporal variation in soil inorganic N

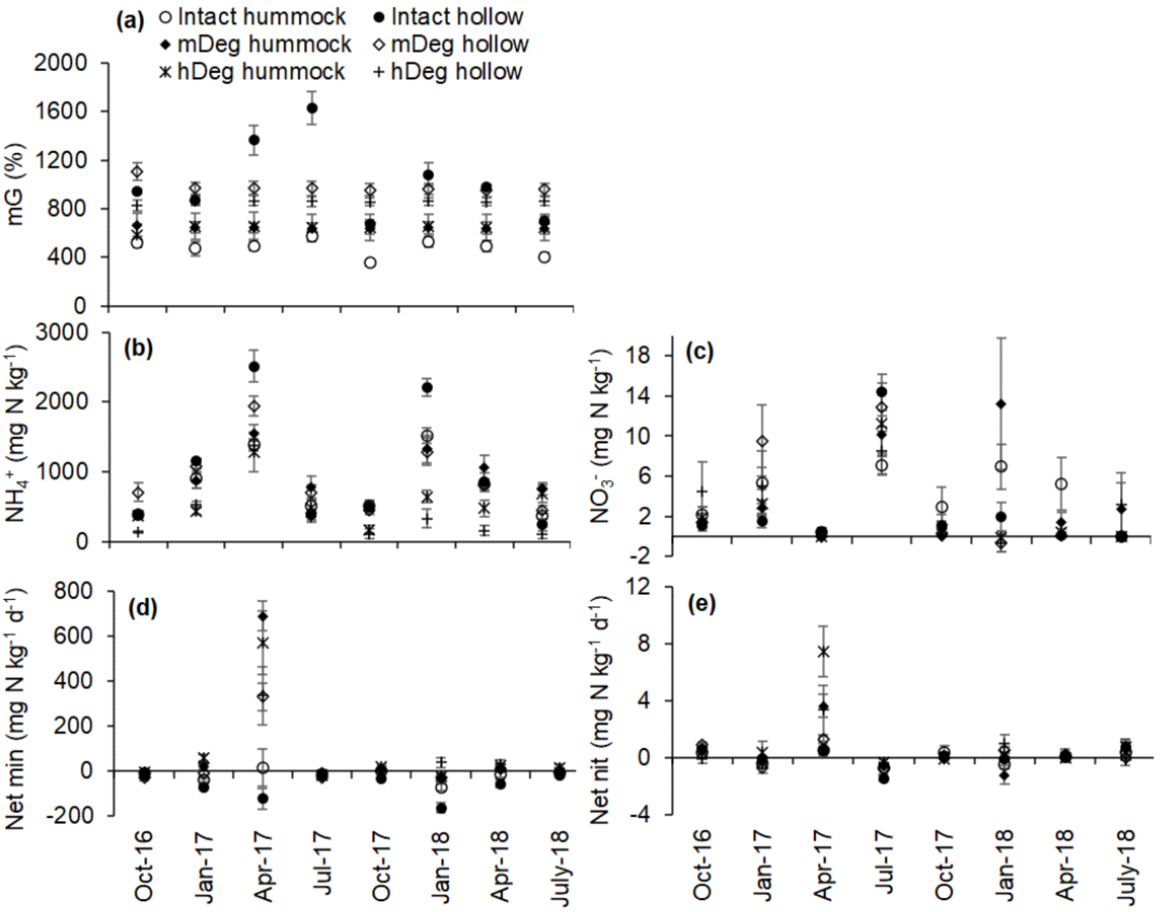

Figure S2: Monthly mean gravimetric moisture (m_G) (a), inorganic N pools (NH_4^+, NO_3^-) (b) and (c), net mineralization (Net min) (d) and net nitrification (Net nit) (e) rates in hummocks and hollows around live palms at the Intact, moderately (mDeg) and heavily (hDeg) degraded sites. Error bars are SE.
